# MegaLTR: a web server and standalone pipeline for detecting and annotating LTR-retrotransposons in plant genomes

**DOI:** 10.3389/fpls.2023.1237426

**Published:** 2023-09-20

**Authors:** Morad M. Mokhtar, Achraf El Allali

**Affiliations:** African Genome Center, Mohammed VI Polytechnic University, Benguerir, Morocco

**Keywords:** LTR-retrotransposons, plant genomes, webserver, insertion age, LTR-RT gene chimeras, non-redundant LTR-RTs library

## Abstract

LTR-retrotransposons (LTR-RTs) are a class of RNA-replicating transposon elements (TEs) that can alter genome structure and function by moving positions, repositioning genes, shifting exons, and causing chromosomal rearrangements. LTR-RTs are widespread in many plant genomes and constitute a significant portion of the genome. Their movement and activity in eukaryotic genomes can provide insight into genome evolution and gene function, especially when LTR-RTs are located near or within genes. Building the redundant and non-redundant LTR-RTs libraries and their annotations for species lacking this resource requires extensive bioinformatics pipelines and expensive computing power to analyze large amounts of genomic data. This increases the need for online services that provide computational resources with minimal overhead and maximum efficiency. Here, we present MegaLTR as a web server and standalone pipeline that detects intact LTR-RTs at the whole-genome level and integrates multiple tools for structure-based, homologybased, and *de novo* identification, classification, annotation, insertion time determination, and LTR-RT gene chimera analysis. MegaLTR also provides statistical analysis and visualization with multiple tools and can be used to accelerate plant species discovery and assist breeding programs in their efforts to improve genomic resources. We hope that the development of online services such as MegaLTR, which can analyze large amounts of genomic data, will become increasingly important for the automated detection and annotation of LTR-RT elements.

## 1 Introduction

Long Terminal Repeat (LTR) Retrotransposons (LTR-RTs) are a class of transposon elements (TEs) belonging to the repetitive DNA sequences that have played a crucial role in shaping the structure and function of eukaryotic genomes (Vitte and Panaud, 2005). LTR-RTs are characterized by their ability to move within genomes *via* a “copy-and-paste” mechanism that involves transcription into RNA, reverse transcription into DNA, and subsequent insertion into new genomic locations (Lopes et al., 2013). These elements have been found in various organisms, including plants, where they contribute significantly to genome size and complexity. LTR-RTs are of great interest in the field of genomics because of their importance in genome evolution, gene regulation, and understanding plant biology (Bennetzen and Wang, 2014). Plant genomes are often characterized by a high proportion of TEs, with LTR-RTs being one of the major contributors to these elements. TEs can make up a substantial portion of the plant genome, as in maize, where TEs account for 85% of the genome, of which LTR-RTs account for 75% (Schnable et al., 2009). This wide distribution highlights their importance in shaping genome architecture and dynamics (Schnable et al., 2009). LTR-RTs are known to play a role in creating genetic diversity, promoting chromosomal rearrangements and influencing gene expression through their insertion sites and regulatory sequences (Bennetzen and Wang, 2014). Therefore, the study of LTR-RTs is crucial to unravel the complexity of plant genomes and understand their functional implications (Xia et al., 2020). The study of LTR-RTs provides insights into various aspects of plant genome biology. For example, studying their structural diversity, insertion patterns, and distribution in plant taxa can provide insight into evolutionary history and interspecies relationships (Grandbastien et al., 2005). In addition, understanding the regulation of LTR-RTs activity and its interplay with host factors can provide insight into the mechanisms of genome stability (Vitte et al., 2014). Because LTR-RTs can influence nearby gene expression through epigenetic modifications and transcriptional interference, studying these elements contributes to our understanding of gene regulatory networks in plants (Zhao et al., 2016; Mokhtar et al., 2021).

The movement of LTR-RTs within genomes contributes to genome evolution by generating genetic variation and driving genome expansion (Vitte et al., 2014). These elements can facilitate chromosomal rearrangements through unequal homologous recombination between LTRs or ectopic recombination between non-homologous LTRs. Such events can lead to gene duplications, deletions, and chromosomal rearrangements that contribute to plant genome diversification (Ma et al., 2004). LTR-RTs may also serve as targets for silencing by small RNAs, which could affect their transposition rates and influence the evolutionary development of plant species (Franco-Zorrilla et al., 2007). While some LTR-RTs are likely to be transcriptionally inactive, accumulating evidence suggests that many elements have been co-opted for useful functions in plant genomes. For example, some LTR-RTs have been domesticated to provide regulatory sequences such as promoters and enhancers for nearby genes (Jung et al., 2019). In addition, they have been associated with stress responses, chromatin remodeling, and even symbiotic interactions (Ito et al., 2016; Pereira, 2016). Understanding the functional significance of LTR-RTs in plant genomes provides insights into the intricate interplay between repetitive DNA elements and the evolution of novel traits.

LTR-RTs consist of several different structural elements that play different roles in the movement and regulation of the element within the genome. Common elements include target site duplication (TSD), two semi-identical LTRs, polypurine tract (PPT), primer binding site (PBS), *GAG* and *Pol* genes (Kumar, 1998). LTRs are long stretches of DNA located at both ends of the element and are typically several hundred base pairs long. LTRs contain regulatory elements (promoters, enhancers) and are thought to be important for the integration and stability of the element in the genome (Kumar, 1998). *GAG* and *Pol* genes are genes that encode proteins involved in the movement and replication of the element (Eickbush and Jamburuthugoda, 2008). The *GAG* gene encodes a structural protein involved in the assembly of the element, while the *Pol* gene consists of several different functional domains, including protease (PROT), reverse transcriptase (RT), RNase H (RH), and integrase (INT) (Ustyantsev et al., 2015).The RT domain is responsible for synthesizing a DNA copy of the RNA template of the element, while the INT domain is responsible for integrating the element into the genome (Zhao et al., 2016). The PROT domain is responsible for cleavage of the *Pol* protein into its functional domains; the RH domain is involved in degradation of the RNA template during reverse transcription; and other domains that are involved in various aspects of movement and regulation of the element (Gao et al., 2003; Ustyantsev et al., 2015). LTR-RTs are divided into two main categories based on their mode of movement: autonomous and non-autonomous. Autonomous LTR-RTs are capable of moving by themselves, whereas non-autonomous LTR-RTs require the assistance of an autonomous element to move (Wicker et al., 2007). In addition, LTR-RTs are classified into superfamilies *Copia* and *Gypsy* based on internal domain arrangements (Wicker et al., 2007). Other LTR-RTs groups include *LARD* (LArge Retrotransposon Derivatives), *BARE-2* (Barley RetroElement-2), *TR-GAG* (Terminal Repeat Retrotransposons with *GAG* domain), and *TRIM* (Terminal Repeats In Miniature)((Witte et al., 2001; Kalendar et al., 2004; Tanskanen et al., 2007; Chaparro et al., 2015), respectively).

Despite their widespread use and importance, LTR-RTs remain difficult to identify and annotate in most non-model organisms (Ou et al., 2019). One reason is that they are often difficult to identify and track in the genome. They are also difficult to study because they have complex and variable structures and can interact in complex ways with other DNA sequences (Ou et al., 2019). However, research on LTR-RTs has increased in recent years, thanks to advances in sequencing technology and bioinformatics that have improved our understanding of the role of LTR-RT in genomes. Several tools, pipelines, and databases exist to identify LTR-RTs and support current and future functional genomics research. These tools include Tandem Repeats Finder [TRF, (Benson, 1999)], LTR_STRUC (McCarthy and McDonald, 2003), LTR_FINDER (Xu and Wang, 2007), LTRdigest (Steinbiss et al., 2009), LTRharvest (Ellinghaus et al., 2008), RepeatMasker (Smit et al., 2015), MGEScan3 (Lee et al., 2016), LTR_retriever (Ou and Jiang, 2017), LtrDetector (Valencia and Girgis, 2019), DARTS (Biryukov and Ustyantsev, 2021), and TEsorter (Zhang et al., 2022). Once LTR-RTs are identified, they can be annotated using various databases and resources. Some examples of databases and resources developed for this purpose are TREP (Wicker et al., 2002), RepBase (Jurka et al., 2005), REXdb (Neumann et al., 2019), PlantRep (Amselem et al., 2019), and PlantLTRdb (Mokhtar et al., 2023b). These tools and databases have been used to create automatized pipelines for LTR-RT analysis, including REPCLASS (Feschotte et al., 2009), EDTA (Ou et al., 2019), and Inpactor2 (Orozco-Arias et al., 2022).

EDTA is a pipeline that integrates structural-, homology-based, and *de novo* identification methods to create TEs libraries. EDTA combines LTRharvest, LTR_FINDER, and LTR_retriever to analyze LTR-RTs. In addition, Generic Repeat Finder (Shi and Liang, 2019), TIR-Learner (Su et al., 2019), HelitronScanner (Xiong et al., 2014), and RepeatModeler (Smit et al., 2015) are used for other TEs. For LTR-RTs, EDTA performs identification, superfamily-level classification (*Copia* and *Gypsy*), and insertion age estimation with highly efficient tools. Another available pipeline is Inpactor2. It integrates the process of identification and classification of LTR-RTs at the lineage level and runs in a reasonable time. While EDTA and Inpactor2 are comprehensive pipelines for creating LTR-RTs libraries, it lacks some features, such as putative autonomous and non-autonomous classification, identification of LTR-RT gene chimeras, detection of LTR-RTs near genes, statistical analysis and visualization of LTR-RTs, and adjustable parameters for each analysis step. It is also not available as a web server and requires some level of technical computer skills. Like any machine learning-based algorithm, Inpactor2 is dependent on the quality of its training dataset (Orozco-Arias et al., 2022), a fact that users should consider when using this algorithm.

Here we introduce MegaLTR as a web server and standalone pipeline that detects intact LTR-RTs at the whole genome level. MegaLTR integrates multiple tools for structure-based, homology-based, and *de novo* identification, classification, and annotation. MegaLTR performs classification into putative autonomous and non-autonomous, superfamilial and lineage levels. It also identifies LTR-RT gene chimeras, detects LTR-RTs near genes, statistical analysis and visualization of LTR-RT. MegaLTR is easy to use and allows customization of parameters for each analysis step in both its web server and standalone versions.

## 2 Materials and methods

### 2.1 Genomic data

The complete genome sequences and annotations of 26 plant species were downloaded from the NCBI database (Wheeler et al., 2007). These genomes were selected based on some criteria, such as annotation and LTR assembly index (LAI) score (Ou et al., 2018), genome size, number of pseudomolecules/scaffolds, and the fact that they were model and non-model plants. The LAI score has been widely used in recent years to assess the quality of genome assemblies. It has been shown to be useful in determining the quality of assemblies, as a higher LAI score is associated with a higher quality assembly (Ou et al., 2018). The LAI score of each species was taken from the PlantLAI database (Mokhtar et al., 2023a). The plant name, NCBI taxonomy ID, GenBank accession number, assembly level, LAI score, genome size, evolutionary rate, and number of pseudomolecules/scaffolds of the studied species are listed in **Table S1**.

### 2.2 MegaLTR design and workflow

**Figure 1:**
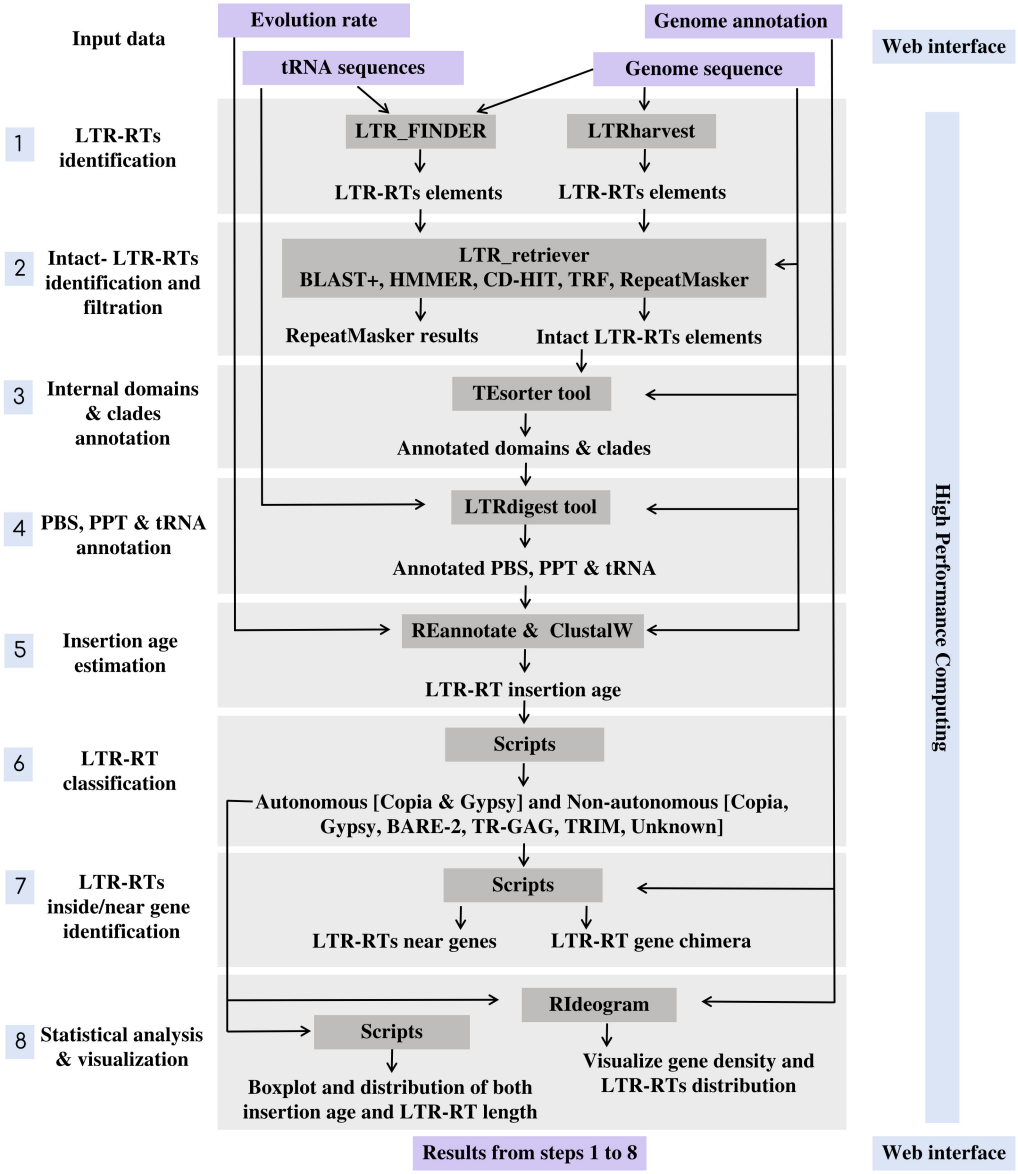
An Overview of MegaLTR Workflow and procedure.

MegaLTR’s workflow includes multiple programs interconnected by data adapters to ensure that data is routed from the server to a high-performance computer (HPC) and back to the server and processed as an end-to-end pipeline. The implementation of MegaLTR was summarized in **Data Sheet 1**. The MegaLTR workflow is shown schematically in **Figure 1**. MegaLTR is designed to accept FASTA sequences and their GFF annotation as input. It is capable of processing whole genome sequences in any form, including chromesomes, pseudomolecules, scaffolds, contigs, and fragments, which is useful in draft genome analysis. Analysis with MegaLTR consists of eight main steps: 1) LTR-RTs identification with LTR_FINDER (Xu and Wang, 2007; Ou and Jiang, 2019) and LTRharvest (Ellinghaus et al., 2008); 2) filtering LTR-RTs with LTR_retriever (Ou and Jiang, 2017); 3) annotation of internal domains and clades with TEsorter (Zhang et al., 2022); 4) PBS and PPT annotation with LTRdigest (Steinbiss et al., 2009) and PltRNAdb (Mokhtar and El Allali, 2022); 5) insertion age estimation with REANNOTATE (Pereira, 2008) and ClustalW (Thompson et al., 2003); 6) LTR-RTs classification with Python scripts and create a non-redundant LTRRTs library using USEARCH v11.0 (Edgar, 2010); 7) LTR-RTs detection within and near genes with Perl scripts; 8) statistical analysis and visualization with Python, R scripts and RIdeograms (Hao et al., 2020). The user can set the parameters for each analysis step.

For identification of LTR-RT candidates, LTR_FINDER and LTRharvest are used because they are very effective in identifying LTR-RTs and outperform all other programs in sensitivity (Ou and Jiang, 2017). However, these programs tend to produce a number of false-positive predictions (Lerat, 2010). To effectively remove false-positive predictions made by the original softwares, the results were combined into one file and used as input to LTR_retriever. The LTR_retriever tool uses a combination of several programs, including HMMER (Wheeler and Eddy, 2013), CD-HIT (Li and Godzik, 2006), BLAST+ (Camacho et al., 2009), RepeatMasker (Smit et al., 2015), and TRF (Benson, 1999) to identify and filter out all false candidates for LTR-RTs. MegaLTR only considers intact LTR-RT candidates that pass these filtering steps in the post analysis. The intact LTR-RT, defined as candidates, contain two identical/semi-identical LTRs and a target site duplication at both ends. The LTRs contain conserved sequences such as the TG-CA, which may play a role in regulating retrotransposon expression and/or retrotransposition. To accurately identify features within a potential LTR-RT, MegaLTR uses LTRdigest to detect PPT, and PBS and TEsorter to analyze internal protein domains. The PBS is generally located near the 5’LTR, while PPT is relatively close to the 3’LTR. To identify the PBS, a tRNA sequence library is used to search for regions in the LTR-RT candidate that are complementary to the tRNA. The tRNA sequences for this analysis are from the plant tRNA database [PltRNAdb, (Mokhtar and El Allali, 2022)]. This procedure allows reliable identification of PBS and PPT within a LTR-RT candidate. To annotate protein domains, TEsorter searched one of the databases REXdb (Neumann et al., 2019) and GyDB (http://gydb.org) using HMMScan (Eddy, 1998) to identify putative domains such as capsid protein, protease, reverse transcriptase, RNase H, and integrase.

The next step is to classify LTR-RTs in clades. Previous studies have proposed different clade-level classifications for LTR-RTs. Neumann et al. (2019) divided *Copia* to the clades *Ale, Alesia, SIRE, Bianca, Lyco, Ikeros, Gymco I-IV, Bryco, Osser, TAR, Angela, Ivana,* and *Tork*. They also divided *Gypsy* into the clades *Chlamyvir, CRM, Tcn1, Reina, Galadriel, Tekay, Tat-I-III, Athila, Ogre, Phygy, Selgy*, and *Retand*. This classification is based on the protein domain databases for clade-level classification of LTR-RT. The TEsorter tool uses these databases as well as REXdb and GyDB to classify LTR-RTs into superfamilies and further classify them into clades. To estimate the insertion age of LTR-RT, MegaLTR uses the tools REANNOTATE and ClustalW in combination to estimate the insertion age of LTR-RT elements based on a comparative analysis of their 5’ and 3’ LTRs. To calculate the insertion age, the Kimura-2 parameter model (Kimura, 1980) is used to calculate the substitutions per site rate (K) between LTRs. The age is then estimated as T= K/2r (Kimura, 1980), where (r) is the evolution rate. In MegaLTR, the evolution rate is usually set by the user. It is important to note that evolution rates can vary significantly between species.

**Figure 2:**
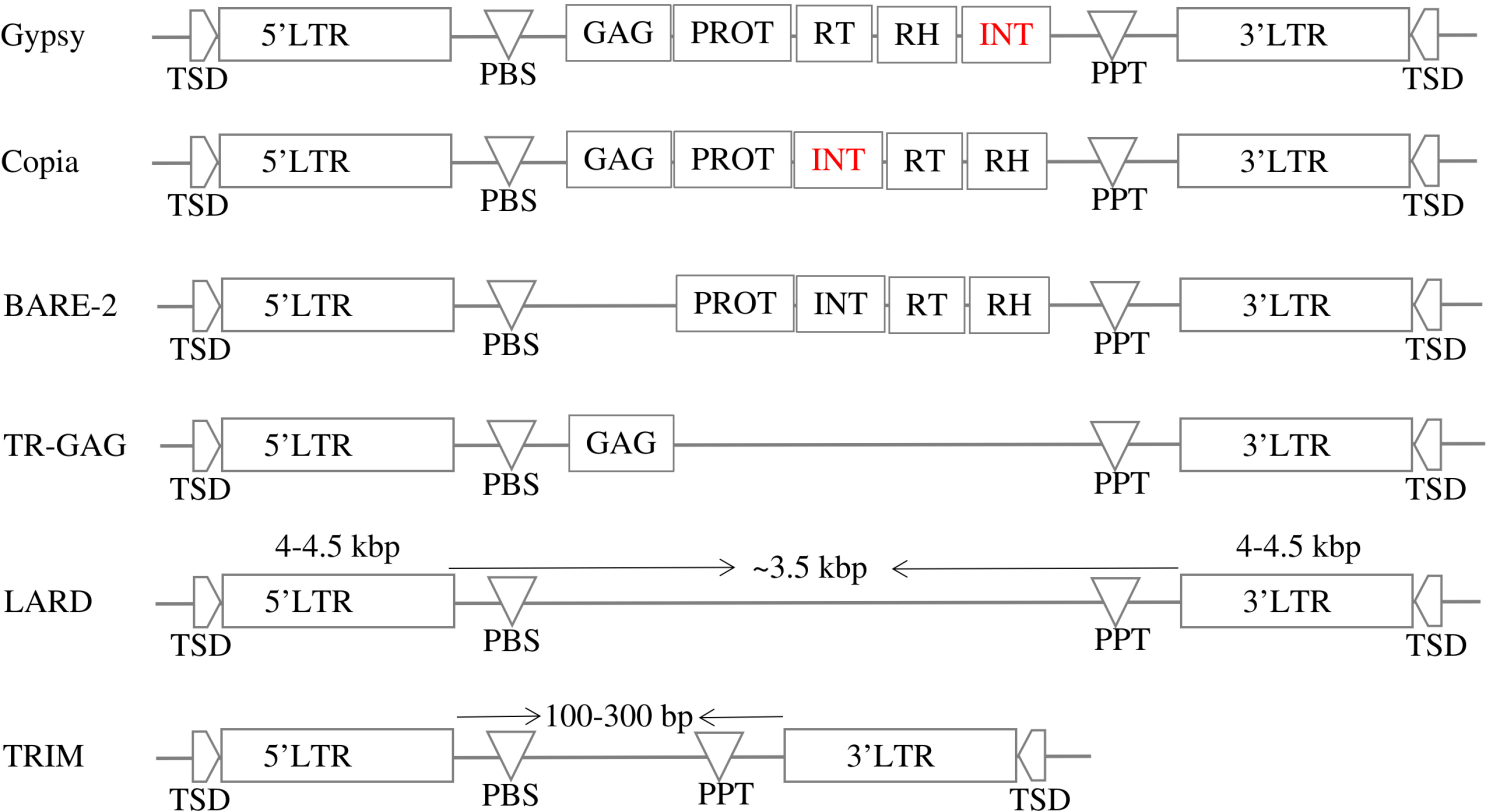
The structures of autonomous (*Gypsy* and *Copia*) and non-autonomous LTR-RTs (*LARD, TRIM, TR-GAG*, and *BARE-2*).

LTR-RT can be divided into two main categories based on their structure: autonomous and nonautonomous. According to Wicker et al. (2007), the structure of autonomous *Gypsy* and *Copia* is based on domains arranged within the element LTR-RT. The structure of *Gypsy* is TSD-LTR-PBS-GAG-PROT-RTRH-INT-PPT-LTR-TSD, while *Copia* is TSD-LTR-PBS-GAG-PROT-INT-RT-RH-PPT-LTR-TSD. *Copia* and *Gypsy* elements that no longer have any of the previous structures are classified as non-autonomous Copia and non-autonomous *Gypsy*. Non-autonomous LTR-RT can be further subdivided based on their specific structure and the presence or absence of certain domains. Examples of non-autonomous elements include *LARD, TRIM, TR-GAG*, and *BARE-2* (**Figure 2**). The specific criteria for classifying LTR-RT elements into these categories have been described in several research studies, including Kalendar et al. (2004); Witte et al. (2001); Chaparro et al. (2015); Tanskanen et al. (2007). LTR-RT elements that do not fit into any of these categories and are not classified as autonomous or non-autonomous *Copia* or *Gypsy* elements are classified as “unknown”.

Because LTR-RTs sometimes insert themselves into or near genes and can affect gene function. MegaLTR identifies LTR-RTs that are inside or near genes using Perl scripts. To classify LTR-RT elements based on their genomic location, the start and end coordinates of the gene and the start and end coordinates of the LTR-RT element within the genome can be compared. If the LTR-RT element is located within the coordinates of the gene, it is considered a gene chimera. If the LTR-RT element is located near a gene, the distance upstream and downstream of the LTR-RT element can be determined in base pairs. This distance is usually determined by the user and may vary depending on the specific research question and desired sensitivity for detecting LTR-RT elements near genes. In the final step, MegaLTR performs two statistical analyses using boxplot. One for LTR-RT length by bps and the other for LTR-RT insertion age. Boxplots are useful for quickly conveying information about the variability and skewness of a data set. The next step is a visualization of the distribution of the identified LTR-RT and gene density in each pseudomolecules/scaffolds using RIdeograms (Hao et al., 2020).

### 2.3 Standalone version

The standalone version of MegaLTR is also available (https://github.com/MoradMMokhtar/MegaLTR). It has been thoroughly tested on Ubuntu 18.04 and 20.04. Installation is effortless *via* a Conda environment with the command: conda env create -f MegaLTR.yml. This command not only installs MegaLTR, but also takes care of installing the associated dependencies. Using MegaLTR standalone, the user can define all parameters using the following flags: -A (the analysis type), -F (fasta file), -G (GFF file), -T (species name for tRNA database), -P (prefix for outfiles), -l (minimum length of 5’ & 3’LTR, -L (maximum length of 5’ & 3’LTR), -d (minimum distance between 5’ & 3’LTR), -D (maximum distance between 5’ & 3’LTR), -S (similarity threshold), -M (minimum length of exact match pair), -B (name of TE database that TEsorter will use “gydb, rexdb, rexdb-plant, rexdb-metazoa”), -C (minimum coverage for protein domains in HMMScan), -V (maximum E value for protein domains in HMMScan), -Q (classification rule [identity - coverage - length]), -E (hmm database), -R (mutation rate of neutral species), -U (distance upstream LTR-RTs to determine nearby genes), -X (distance downstream LTR-RTs), -W (gene density window size), -N (number of chromosomes), -t (number of CPUs to run MegaLTR).

## 3 Results and discussion

### 3.1 Validation and comparison

To test the performance and validate the quality of the intact LTR-RTs identified by MegaLTR, a manual curation of LTR-RTs library from *Oryza sativa* was used to compare the non-redundant library generated by MegaLTR. The curated *Oryza sativa* library included 897 LTR-RT elements and was previously established by Ou and Jiang (2017). RepeatMasker v4.0.7 with the parameters “-e ncbi -pa 56 -no_is -q -norna -div 40 -nolow -lib [LTR -library] -cutoff 225 genome.fa” was applied to the MegaLTR library and the curated library to compute the performance metrics. We used six metrics proposed by Ou and Jiang (2017) to characterize the annotation performance of the non-redundant LTR-RT library generated by MegaLTR. These metrics include sensitivity (the ability to annotate target sequences correctly), specificity (the ability to exclude non-target sequences correctly), accuracy (true discrimination rate between target and non-target sequences), precision (true detection rate), FDR (false detection rate), and F1 measure (harmonic mean of precision and sensitivity). The True-positives (TP), false-positives (FP), false-negatives (FN), and true-negatives (TN) rates were computed using the EDTA toolkit. The performance metrics are defined as:


$$\begin{array}{ccc} Sensitivity=\frac{TP}{TP+FN} & Specificity=\frac{TN}{FP+TN} & Accuracy=\frac{TP+TN}{TP+TN+FP+FN}\\ \;Precision=\frac{TP}{TP+FP} & F1=\frac{2*TP}{2*TP+FP+FN} & FDR=1-\frac{TP}{TP+FP} \end{array}$$

MegaLTR results show consistently high specificity (96.59%), accuracy (94.98%), precision (89.38%), sensitivity (89.92%), and F1 measure (89.65%). The relatively low FDR (10.61%) confirms the accuracy and reliability of the LTR-RTs identified by MegaLTR. For comparison purposes, the EDTA pipeline was used to analyze the whole genome of *Oryza sativa* using the same parameters used in MegaLTR (-D 15000 -d 1000 -L 7000 -l 100 -p 20 -M 0.85). The EDTA-generated LTR-RTs library was compared with the curated *Oryza sativa* LTR library. Similar to the evaluation of MegaLTR, RepeatMasker and the script “lib-test.pl” were used to calculate the evaluation metrics. The results of the EDTA metrics were: specificity (96.23%), accuracy (94.61%), precision (88.34%), sensitivity (89.52%), F1 measure (88.93%), and FDR (11.65%). As shown in **Table 1**, MegaLTR has relatively higher specificity, accuracy, precision and sensitivity with low FDR compared to EDTA.

**Table 1:** Comparison of six metrics between MegaLTR and EDTA using the genome of *Oryza sativa*.

Pipeline name	Sensitivity	Specificity	Accuracy	Precision	FDR	F1
MegaLTR	89.92%	96.59%	94.98%	89.38%	10.61%	89.65%
EDTA	89.52%	96.23%	94.61%	88.34%	11.65%	88.93%

Overall, the comparison of MegaLTR with both the manually curated *Oryza sativa* library and EDTA demonstrates the robustness and effectiveness of MegaLTR in identifying intact LTR-RTs and provides valuable insights for future studies on retrotransposons in plant genomes. **Table 2** shows a comparison of various features between the MegaLTR and EDTA. The features compared include the class of TEs identified, the level of classification (autonomous, non-autonomous, superfamily, lineage level), the identification of LTR-RT near and within genes, and the form of availability.

**Table 2:** Comparison of some features between MegaLTR and EDTA.

	Identified TEs		Classification level			Identify LTR-RT		Availability		
	DNA TEs	LTR-RTs	Autonomous and non-autonomous	Superfamily	Lineage level	Gene chimeras	Near genes	Web server	Standalone	
MegaLTR	X	✓	✓	✓	✓	✓	✓	✓	✓	
EDTA	✓	✓	X	✓	X	X	X	X	✓	
LTR-RTs sub-classification level										
	***Copia***	***Gypsy***	**Unknown**	***LARD***	***TRIM***	***TR-GAG***	***BARE-2***			
MegaLTR	✓	✓	✓	✓	✓	✓	✓			
EDTA	✓	✓	✓	X	X	X	X			

"✓" refer to the feature is found, and "X" refers to the feature is missing.

To validate MegaLTR, 26 whole-genome sequences with a total volume of 15.33 Gbp representing 58,392 scaffolds/pseudomolecules were used. Plant species were selected based on their LAI score, genome size, and number of scaffolds/pseudomolecules. As suggested by Ou et al., (2018), the LAI score for draft genomes is below 10, while reference genomes have a LAI score between 10 and 20. Gold genomes have LAI scores greater than 20. The LAI scores of the selected genomes were retrieved from the PlantLAI database (Mokhtar et al., 2023a) and ranged from 8.7 (*Citrus unshiu*) to 29.45 (*Zea mays*), covering the different qualities of genome sequences (draft, reference, and gold quality). Genome sizes also varied, ranging from 119.6 Mbp for *Arabidopsis thaliana* to 2182.79 Mbp for *Zea mays*. In addition, the number of scaffolds/pseudomolecules varied from 7 to 20,876 for *Arabidopsis thaliana* and *Citrus unshiu* (**Table S1**).

**Table 3** shows a comparison between MegaLTR and EDTA based on runtime and number of identified LTR-RTs in each classified superfamily using the same parameters mentioned above. Since EDTA performs the analysis of all TEs (LTR, TIR, and Helitron), we used the LTR [–type ltr] option to analyze only the LTR-RTs candidates. For MegaLTR, the total number of identified autonomous (*Gypsy* and *Copia*) and nonautonomous LTR-RTs (*Gypsy, Copia, BARE-2, TR-GAG*, unknown) was reported for the genomes examined. The *LARD* and *TRIM* structures were not detected in these genomes. However, EDTA classified LTR-RTs into *Gypsy, Copia* and unknown elements. As can be seen in **Table 3**, MegaLTR reported a small number of unknown elements compared to EDTA, as MegaLTR performed further analyses to annotate and classify the identified LTR-RTs.

**Table 3:** Analysis runtime in hours and minutes (h:m), the total number of identified LTR-RTs in each classified superfamily for the 26 plant species using MegaLTR and EDTA.

Species name	Run time		MegaLTR							EDTA		
	MegaLTR	EDTA	Autonomous				Nonautonomous					
			*Gypsy*	*Copia*	*Gypsy*	*Copia*	*BARE-2*	*TR-GAG*	unknown	*Gypsy*	*Copia*	unknown
*Arabidopsis thaliana*	0:09	0:14	2	–	118	80	–	1	2	105	75	27
*Brassica rapa*	0:48	0:38	189	65	1138	1238	34	18	228	1196	1074	588
*Citrus clementina*	0:15	0:24	60	23	771	846	1	20	14	820	765	62
*Citrus unshiu*	0:17	0:20	26	4	340	178	–	2	2	343	161	32
*Cucumis sativus*	0:09	0:06	31	7	135	219	2	–	22	159	196	73
*Glycine max*	0:54	1:13	227	69	2046	2335	22	322	145	2433	1388	694
*Medicago truncatula*	0:26	0:31	25	21	743	1385	2	–	130	710	1329	335
*Mikania micrantha*	4:51	14:43	470	145	6930	15356	9	58	705	7009	14100	2671
*Oryza sativa Japonica*	0:28	0:25	35	9	488	1399	4	4	168	502	1504	226
*Panicum hallii*	1:03	1:23	46	46	841	3539	1	3	293	866	3512	641
*Phoenix dactylifera*	1:21	2:29	498	226	6233	3016	2	69	148	6625	2176	503
*Physcomitrella patens*	0:21	0:27	–	–	184	3225	–	–	13	140	3069	122
*Populus trichocarpa*	0:18	0:26	59	19	501	474	–	1	59	523	448	170
*Prunus persica*	0:16	0:30	43	21	632	326	5	1	141	637	319	548
*Rosa chinensis*	0:44	1:43	113	20	3806	1614	38	21	745	3498	1426	1884
*Salvia splendens*	2:16	3:18	198	296	4183	5687	23	70	459	3898	5462	2009
*Selaginella moellendorffii*	0:11	0:10	–	102	26	557	1	7	337	34	627	355
*Sesamum indicum*	0:21	0:28	5	20	258	176	1	6	6	240	185	38
*Setaria viridis*	0:25	0:36	3	39	829	1071	1	5	10	802	1091	105
*Solanum lycopersicum*	0:32	0:35	93	15	945	774	–	5	30	899	719	196
*Solanum pennellii*	0:37	0:42	143	1	1714	443	1	6	58	1622	361	310
*Sorghum bicolor*	1:28	2:35	58	109	1096	7779	3	7	893	1167	6927	2120
*Trifolium pratense*	0:38	1:00	83	95	2692	1566	5	34	943	2470	1469	2074
*Vitis vinifera*	0:22	0:35	247	10	1090	613	25	1	64	1275	583	247
*Zea mays*	10:16	27:39	3687	191	16301	28080	70	496	3163	19836	26376	6221

The EDTA runtime given in **Table 3** refers to LTR-RTs identification and classification as given by EDTA. However, the runtime given by MegaLTR refers to all analyses, including identification, annotation, classification of LTR-RTs, identification of LTR-RT gene chimeras, detection of LTR-RTs near genes, statistical analysis, and visualization of the density of LTR-RTs. The run times of each step reported by EDTA and MegaLTR can be found in **Data Sheet 3** and **Data Sheet 4**, respectively. For MegaLTR, analysis times range from 9 minutes for *Arabidopsis lyrata* (206.8 Mb), *Arabidopsis thaliana* (119.6 Mb), and *Cucumis sativus* (226.6 Mb) to 10 hours and 16 minutes for *Zea mays* (2182.7 Mb). For EDTA, analysis times range from 6 minutes for *Cucumis sativus* to 27 hours and 39 minutes for *Zea mays*. For large genomes such as *Zea mays* (2182.7 Mb) and *Mikania micrantha* (1790.6 Mb), MegaLTR is more than 2x faster than EDTA. **Figure S1** shows that MegaLTR is faster than EDTA for large genomes. The total number of LTR-RTs identified is also similar and only slightly different between MegaLTR and EDTA (**Figure S1**).

### 3.2 Case study: *Arabidopsis thaliana* genome

*Arabidopsis thaliana* was selected as a case study for MegaLTR results and serves as a comparison between the output of MegaLTR and EDTA in terms of classification. *Arabidopsis thaliana* is a model organism with a well-structured genome arranged in chromosomes and a high LAI score of 16.91. EDTA identified a total of 207 intact LTR-RTs elements, including 105 *Gypsy*, 75 *Copia*, and 27 unknown. For MegaLTR, a total of 203 intact LTR-RT elements were identified, classified, and annotated. Of the 203 intact LTR-RTs elements, 2 elements were classified as autonomous *Gypsy* and 201 as non-autonomous LTR-RTs. Non-autonomous elements included 118 *Gypsy*, 80 *Copia*, 1 *TR-GAG*, and 2 unknown (**Table 3**). Based on the position of the identified elements in the genome sequence, the LTR-RT results of EDTA and MegaLTR were compared. Of the 207 LTR-RTs identified by EDTA, 193 elements matched MegaLTR and 14 did not match. These 14 LTR-RTs included one element that did not pass the LTR_retriever filter and 11 elements that did not pass the TEsorter filter in MegaLTR. EDTA assigned these 11 elements to the NA class, consistent with their exclusion by MegaLTR. The remaining 2 elements were not found in the MegaLTR data. On the other hand, MegaLTR identified 10 LTR-RTs not found by EDTA (**Figure S2A**), including 7 *Gypsy* and 3 *Copia*. These elements are assigned to 7 clades, including 3 *Athila*, 2 *Retand*, 1 *Ale*, 1 *Ivana*, 1 *Reina*, 1 *SIRE*, and 1 *Tekay*. As for the internal domains, 6 elements contain all the domains necessary for transposition (*GAG*, PROT, INT, RT, RH) for Copia and (*GAG*, PROT, RT, RH, INT) for *Gypsy*. The remaining 4 elements include one element containing the domains *GAG* and PROT, 2 elements containing the domain PROT, and one containing the domain *GAG*. The annotation of MegaLTR’s unique results suggests that MegaLTR is able to identify more intact LTR-RTs with a high degree of filtering and annotation. In contrast, EDTA reported a number of elements that do not belong to LTR (**Data Sheet 5**).

MegaLTR is able to classify intact LTR-RT elements into autonomous (*Gypsy*) and non-autonomous (*Copia, Gypsy*, and *TR-GAG*) based on their structure. In addition, MegaLTR is able to classify unknown elements into superfamilies. EDTA reported 27 unknown elements, while MegaLTR reported only 2 unknown elements. As shown in **Figure (S2B)**, MegaLTR and EDTA have 2 unknown elements in common, while EDTA has 25 unique unknown elements. The 25 unknown elements include 12 elements that did not pass MegaLTR filtering steps and 13 elements that were annotated and classified as nonautonomous (*Copia* and *Gypsy*). **Data Sheet 5** lists the common LTR-RTs, the unique LTR-RTs in EDTA, the unique LTR-RTs in MegaLTR, the unknown elements in EDTA, and the unknown elements in MegaLTR.

### 3.3 Runtime vs. number of CPUs

**Figure 3:**
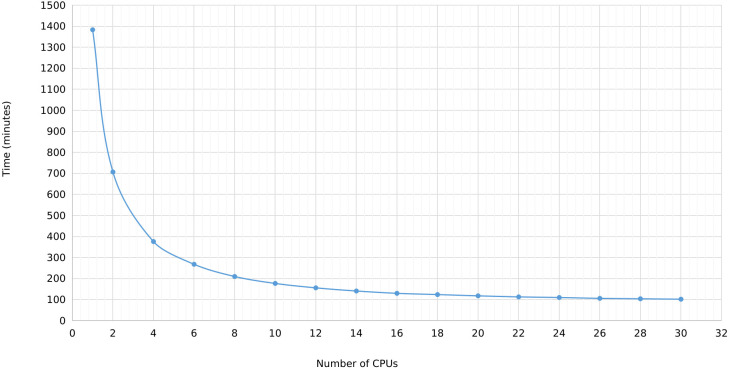
MegaLTR run time of *Brassica rapa* genome using different number of threads ranging from 1 to 30.

In MegaLTR, multithreading was implemented to reduce the execution time. By splitting the genome sequence into scaffold/chromosome without splitting the individual sequences, MegaLTR can analyze multiple sequences simultaneously using multiple CPU cores (threads). In the standalone version, the user can specify the number of threads to use, while the MegaLTR web server currently uses 56 CPU cores for parallel processing. We tested the effect of the number of threads on runtime using the *Brassica rapa* genome. This genome was selected based on its medium genome size (352.9 Mbp) and the number of pseudomolecules/scaffolds (1100). **Figure 3** and **Data Sheet 2** show the runtime for different CPU numbers from 1 to 30. To analyse the Brassica rapa genome using a single thread, MegaLTR required 1382 minutes, while using 2 threads reduced the runtime to 707 minutes and using 30 threads reduced the runtime to 102 minutes, demonstrating the gain achieved through parallel processing in MegaLTR.

### 3.4 Generated output

**Figure 4:**
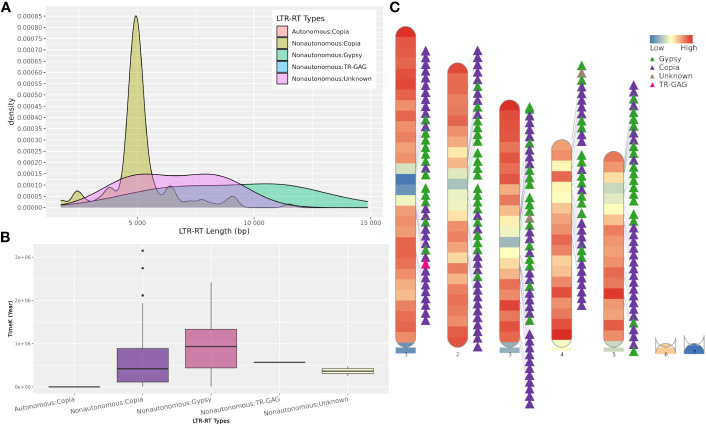
Example of MegaLTR generated results of *Arabidopsis thaliana*. **(A)** LTR-RTs length distribution. **(B)** Boxplot of insertion age, **(C)** Visualization of the density of genes and LTR-RTs on chromosomes.

The web server and standalone version of MegaLTR automatically generate a series of tables, FASTA files, and images, some of which are listed in **Table S2**. These files contain tables with the position of the identified LTR-RT within the sequence, the start and end of all identified features, classification into autonomous, non-autonomous, superfamily and lineage levels, estimated insertion age, LTR-RT-gene chimeras and LTR-RTs-near genes. It also generates redundant and non-redundant LTR-RTs libraries in FASTA format. The full list of generated results can be found in the MegaLTR online documentation (https://github.com/MoradMMokhtar/MegaLTR). Using *Arabidopsis thaliana* genome, **Figure 4** shows an example of statistical analysis of the length of LTR-RT, the age of insertion of LTR-RT, and visualization of the density of genes and LTR-RTs on chromosomes.

## 4 Conclusion and future directions

With the increasing availability of plant genome projects, researchers need accurate, robust, and easy-to-use pipelines for processing large amounts of data to study the effects of LTR-RTs on plant genome evolution and functionality. These pipelines, in the form of a web server, would be valuable for efforts to integrate LTR-RTs as a possible element for studying the gene regulatory system. MegaLTR is a web server and stand-alone pipeline that detects intact LTR-RTs at the whole-genome level and integrates multiple tools for homology-, structure-, and *de novo*-based identification, classification, and annotation of intact LTR-RT. In addition, a comprehensive pipeline is also needed to create a non-redundant library of LTR-RTs for species that lack this resource for annotating whole genome LTR-RTs. MegaLTR is able to classify intact LTR-RT elements into putative autonomous (*Copia* and *Gypsy*) and non-autonomous (*Copia, Gypsy, LARD, TRIM, TR-GAG* and *BARE-2*), superfamily and lineage levels. It also identifies LTR-RT gene chimeras, detects LTR-RTs near genes, and provides statistical analysis and visualization of LTR-RT. For detection of LTR-RTs, MegaLTR shows high specificity, accuracy, precision, sensitivity and low FDR. The development of an online server such as MegaLTR, which provides computational resources for analyzing large amounts of genomic data, is becoming increasingly important for the automated analysis of LTR-RT elements. The current version of MegaLTR focuses on genome-level analysis LTR-RT, with work currently underway to integrate tools optimized for studying LTR-RTs at the transcriptomic level. MegaLTR web server is freely accessible at: https://bioinformatics.um6p.ma/MegaLTR and the standalone version at https://github.com/MoradMMokhtar/MegaLTR.

## Data availability statement

The original contributions presented in the study are included in the article/**Supplementary Material.** Further inquiries can be directed to the corresponding authors. MegaLTR web server is freely available at: https://bioinformatics.um6p.ma/MegaLTR and the standalone version at https://github.com/MoradMMokhtar/MegaLTR.

## Author contributions

Conceptualization MM and AA; Methodology MM and AA; Scripting MM and AA; Data curation MM; Writing–original draft MM and AA. All authors reviewed the manuscript. All authors contributed to the article and approved the submitted version.
