# Supplementary material for: MegaLTR: a web server and standalone pipeline for detecting and annotating LTR-retrotransposons in plant genomes
Source: Front Plant Sci. 2023 Sep 20;14:1237426. doi: 10.3389/fpls.2023.1237426 (PMC10552921; doi:10.3389/fpls.2023.1237426)
Supplement: Supplementary file 1 [file DataSheet_1.docx]

MegaLTR: A web server and standalone pipeline for detecting and annotating LTR-Retrotransposons in plant genomes

**Supplementary File 1: MegaLTR Implementation**

MegaLTR runs on Ubuntu 20.04.3 LTS, Apache (version 2.4.41), PHP (version 7.4.3), MySQL (version 8.0.27), and server (LAMP). Perl (version 5.30.0), Python (version 3.8.10) and R 89 (version 4.1.2) are installed as a prerequisite for the software and tools used in the computation pipeline. The LAMP server runs on a computer with 16-core CPUs, 32 GB of memory, and a 10TB hard drive. Jobs are routed to a large HPC cluster that entered the Top 500 list in the 98th position <https://www.top500.org/system/179908/>
