# Supplementary material for: MegaLTR: a web server and standalone pipeline for detecting and annotating LTR-retrotransposons in plant genomes
Source: Front Plant Sci. 2023 Sep 20;14:1237426. doi: 10.3389/fpls.2023.1237426 (PMC10552921; doi:10.3389/fpls.2023.1237426)
Supplement: Supplementary file 2 [file DataSheet_2.docx]

MegaLTR: A web server and standalone pipeline for detecting and annotating LTR-Retrotransposons in plant genomes

**Supplementary File 2**

**MegaLTR steps with times for the *Brassica rapa* genome using different number of CPUs (1, 2, 4, 6, 8, 10, 12, 14, 16, 18, 20, 22, 24, 26, 28, 30 CPU).**

1 CPU

#############################################

############## MegaLTR v2.0 ##############

#############################################

Contributors: Morad M Mokhtar, Achraf El Allali

Wed Feb 1 00:56:01 +01 2023 Start time

Parameters: -A 3 -F Brassica_rapa.fna -G Brassica_rapa.gff -T Brassica_rapa_trna.fa -P Brassica_rapa_1 -l 100 -L 7000 -d 1000 -D 15000 -S 85 -M 20 -B rexdb -C 20 -V 0.001 -Q 80-80-80 -E rexdb -R 0.000000015 -U 1000 -X 1000 -W 1000000 -N 9 -t 1

Check the FASTA File format.

Wed Feb 1 00:56:08 +01 2023 LTR_FINDER Started

Wed Feb 1 06:55:22 +01 2023 LTR_HARVEST Started

Wed Feb 1 07:14:09 +01 2023 LTR_FINDER & LTR_HARVEST Done

Wed Feb 1 07:14:09 +01 2023 LTR_retriever Started

Wed Feb 1 23:32:16 +01 2023 LTR_retriever Done

Wed Feb 1 23:32:17 +01 2023 LTRdigest Started

Wed Feb 1 23:39:03 +01 2023 LTRdigest Done

Wed Feb 1 23:39:03 +01 2023 TEsorter Started

Wed Feb 1 23:50:32 +01 2023 TEsorter Done

Wed Feb 1 23:50:32 +01 2023 Filtering TEsorter results Started

Wed Feb 1 23:50:32 +01 2023 Mergeing of LTR_retriever, LTRdigest, and TEsorter results

Wed Feb 1 23:54:36 +01 2023 Calculation of the LTR-RT insertion time

Wed Feb 1 23:57:57 +01 2023 Preparing R plots files

Wed Feb 1 23:58:04 +01 2023 LTR-RT insertion time plots done

Wed Feb 1 23:58:05 +01 2023 LTR-RT-gene chimeras started

Wed Feb 1 23:58:21 +01 2023 LTR-RT-gene chimeras done

Wed Feb 1 23:58:21 +01 2023 LTR-RT near genes started

Wed Feb 1 23:58:46 +01 2023 LTR-RT near genes done

Wed Feb 1 23:58:46 +01 2023 Visualization of gene density and LTR-RTs across chromosomes

Wed Feb 1 23:59:39 +01 2023 MegaLTR Done, The results saved in (Brassica_rapa_1/Collected_Files)

1 CPU

#############################################

############## MegaLTR v2.0 ##############

#############################################

Contributors: Morad M Mokhtar, Achraf El Allali

Wed Feb 1 00:56:17 +01 2023 Start time

Parameters: -A 3 -F Brassica_rapa.fna -G Brassica_rapa.gff -T Brassica_rapa_trna.fa -P Brassica_rapa_2 -l 100 -L 7000 -d 1000 -D 15000 -S 85 -M 20 -B rexdb -C 20 -V 0.001 -Q 80-80-80 -E rexdb -R 0.000000015 -U 1000 -X 1000 -W 1000000 -N 9 -t 2

Check the FASTA File format.

Wed Feb 1 00:56:24 +01 2023 LTR_FINDER Started

Wed Feb 1 03:49:28 +01 2023 LTR_HARVEST Started

Wed Feb 1 03:56:01 +01 2023 LTR_FINDER & LTR_HARVEST Done

Wed Feb 1 03:56:01 +01 2023 LTR_retriever Started

Wed Feb 1 12:22:15 +01 2023 LTR_retriever Done

Wed Feb 1 12:22:16 +01 2023 LTRdigest Started

Wed Feb 1 12:28:51 +01 2023 LTRdigest Done

Wed Feb 1 12:28:51 +01 2023 TEsorter Started

Wed Feb 1 12:36:20 +01 2023 TEsorter Done

Wed Feb 1 12:36:20 +01 2023 Filtering TEsorter results Started

Wed Feb 1 12:36:20 +01 2023 Mergeing of LTR_retriever, LTRdigest, and TEsorter results

Wed Feb 1 12:38:28 +01 2023 Calculation of the LTR-RT insertion time

Wed Feb 1 12:41:50 +01 2023 Preparing R plots files

Wed Feb 1 12:41:57 +01 2023 LTR-RT insertion time plots done

Wed Feb 1 12:41:58 +01 2023 LTR-RT-gene chimeras started

Wed Feb 1 12:42:14 +01 2023 LTR-RT-gene chimeras done

Wed Feb 1 12:42:14 +01 2023 LTR-RT near genes started

Wed Feb 1 12:42:42 +01 2023 LTR-RT near genes done

Wed Feb 1 12:42:42 +01 2023 Visualization of gene density and LTR-RTs across chromosomes

Wed Feb 1 12:43:33 +01 2023 MegaLTR Done, The results saved in (Brassica_rapa_2/Collected_Files)

4 CPU

#############################################

############## MegaLTR v2.0 ##############

#############################################

Contributors: Morad M Mokhtar, Achraf El Allali

Wed Feb 1 00:56:32 +01 2023 Start time

Parameters: -A 3 -F Brassica_rapa.fna -G Brassica_rapa.gff -T Brassica_rapa_trna.fa -P Brassica_rapa_4 -l 100 -L 7000 -d 1000 -D 15000 -S 85 -M 20 -B rexdb -C 20 -V 0.001 -Q 80-80-80 -E rexdb -R 0.000000015 -U 1000 -X 1000 -W 1000000 -N 9 -t 4

Check the FASTA File format.

Wed Feb 1 00:56:39 +01 2023 LTR_FINDER Started

Wed Feb 1 02:23:49 +01 2023 LTR_HARVEST Started

Wed Feb 1 02:27:09 +01 2023 LTR_FINDER & LTR_HARVEST Done

Wed Feb 1 02:27:09 +01 2023 LTR_retriever Started

Wed Feb 1 06:55:23 +01 2023 LTR_retriever Done

Wed Feb 1 06:55:24 +01 2023 LTRdigest Started

Wed Feb 1 07:02:00 +01 2023 LTRdigest Done

Wed Feb 1 07:02:00 +01 2023 TEsorter Started

Wed Feb 1 07:06:01 +01 2023 TEsorter Done

Wed Feb 1 07:06:01 +01 2023 Filtering TEsorter results Started

Wed Feb 1 07:06:01 +01 2023 Mergeing of LTR_retriever, LTRdigest, and TEsorter results

Wed Feb 1 07:07:11 +01 2023 Calculation of the LTR-RT insertion time

Wed Feb 1 07:10:32 +01 2023 Preparing R plots files

Wed Feb 1 07:10:39 +01 2023 LTR-RT insertion time plots done

Wed Feb 1 07:10:40 +01 2023 LTR-RT-gene chimeras started

Wed Feb 1 07:10:56 +01 2023 LTR-RT-gene chimeras done

Wed Feb 1 07:10:56 +01 2023 LTR-RT near genes started

Wed Feb 1 07:11:22 +01 2023 LTR-RT near genes done

Wed Feb 1 07:11:22 +01 2023 Visualization of gene density and LTR-RTs across chromosomes

Wed Feb 1 07:12:13 +01 2023 MegaLTR Done, The results saved in (Brassica_rapa_4/Collected_Files)

6 CPU

#############################################

############## MegaLTR v2.0 ##############

#############################################

Contributors: Morad M Mokhtar, Achraf El Allali

Wed Feb 1 00:56:44 +01 2023 Start time

Parameters: -A 3 -F Brassica_rapa.fna -G Brassica_rapa.gff -T Brassica_rapa_trna.fa -P Brassica_rapa_6 -l 100 -L 7000 -d 1000 -D 15000 -S 85 -M 20 -B rexdb -C 20 -V 0.001 -Q 80-80-80 -E rexdb -R 0.000000015 -U 1000 -X 1000 -W 1000000 -N 9 -t 6

Check the FASTA File format.

Wed Feb 1 00:56:51 +01 2023 LTR_FINDER Started

Wed Feb 1 01:55:37 +01 2023 LTR_HARVEST Started

Wed Feb 1 01:57:53 +01 2023 LTR_FINDER & LTR_HARVEST Done

Wed Feb 1 01:57:53 +01 2023 LTR_retriever Started

Wed Feb 1 05:07:29 +01 2023 LTR_retriever Done

Wed Feb 1 05:07:30 +01 2023 LTRdigest Started

Wed Feb 1 05:14:16 +01 2023 LTRdigest Done

Wed Feb 1 05:14:16 +01 2023 TEsorter Started

Wed Feb 1 05:18:13 +01 2023 TEsorter Done

Wed Feb 1 05:18:13 +01 2023 Filtering TEsorter results Started

Wed Feb 1 05:18:13 +01 2023 Mergeing of LTR_retriever, LTRdigest, and TEsorter results

Wed Feb 1 05:19:03 +01 2023 Calculation of the LTR-RT insertion time

Wed Feb 1 05:22:24 +01 2023 Preparing R plots files

Wed Feb 1 05:22:31 +01 2023 LTR-RT insertion time plots done

Wed Feb 1 05:22:32 +01 2023 LTR-RT-gene chimeras started

Wed Feb 1 05:22:49 +01 2023 LTR-RT-gene chimeras done

Wed Feb 1 05:22:49 +01 2023 LTR-RT near genes started

Wed Feb 1 05:23:16 +01 2023 LTR-RT near genes done

Wed Feb 1 05:23:16 +01 2023 Visualization of gene density and LTR-RTs across chromosomes

Wed Feb 1 05:24:08 +01 2023 MegaLTR Done, The results saved in (Brassica_rapa_6/Collected_Files)

8 CPU

#############################################

############## MegaLTR v2.0 ##############

#############################################

Contributors: Morad M Mokhtar, Achraf El Allali

Wed Feb 1 00:56:55 +01 2023 Start time

Parameters: -A 3 -F Brassica_rapa.fna -G Brassica_rapa.gff -T Brassica_rapa_trna.fa -P Brassica_rapa_8 -l 100 -L 7000 -d 1000 -D 15000 -S 85 -M 20 -B rexdb -C 20 -V 0.001 -Q 80-80-80 -E rexdb -R 0.000000015 -U 1000 -X 1000 -W 1000000 -N 9 -t 8

Check the FASTA File format.

Wed Feb 1 00:57:01 +01 2023 LTR_FINDER Started

Wed Feb 1 01:41:27 +01 2023 LTR_HARVEST Started

Wed Feb 1 01:43:11 +01 2023 LTR_FINDER & LTR_HARVEST Done

Wed Feb 1 01:43:11 +01 2023 LTR_retriever Started

Wed Feb 1 04:12:46 +01 2023 LTR_retriever Done

Wed Feb 1 04:12:48 +01 2023 LTRdigest Started

Wed Feb 1 04:19:26 +01 2023 LTRdigest Done

Wed Feb 1 04:19:26 +01 2023 TEsorter Started

Wed Feb 1 04:21:44 +01 2023 TEsorter Done

Wed Feb 1 04:21:44 +01 2023 Filtering TEsorter results Started

Wed Feb 1 04:21:44 +01 2023 Mergeing of LTR_retriever, LTRdigest, and TEsorter results

Wed Feb 1 04:22:24 +01 2023 Calculation of the LTR-RT insertion time

Wed Feb 1 04:25:45 +01 2023 Preparing R plots files

Wed Feb 1 04:25:52 +01 2023 LTR-RT insertion time plots done

Wed Feb 1 04:25:53 +01 2023 LTR-RT-gene chimeras started

Wed Feb 1 04:26:09 +01 2023 LTR-RT-gene chimeras done

Wed Feb 1 04:26:09 +01 2023 LTR-RT near genes started

Wed Feb 1 04:26:35 +01 2023 LTR-RT near genes done

Wed Feb 1 04:26:35 +01 2023 Visualization of gene density and LTR-RTs across chromosomes

Wed Feb 1 04:27:26 +01 2023 MegaLTR Done, The results saved in (Brassica_rapa_8/Collected_Files)

10 CPU

#############################################

############## MegaLTR v2.0 ##############

#############################################

Contributors: Morad M Mokhtar, Achraf El Allali

Wed Feb 1 00:57:07 +01 2023 Start time

Parameters: -A 3 -F Brassica_rapa.fna -G Brassica_rapa.gff -T Brassica_rapa_trna.fa -P Brassica_rapa_10 -l 100 -L 7000 -d 1000 -D 15000 -S 85 -M 20 -B rexdb -C 20 -V 0.001 -Q 80-80-80 -E rexdb -R 0.000000015 -U 1000 -X 1000 -W 1000000 -N 9 -t 10

Check the FASTA File format.

Wed Feb 1 00:57:13 +01 2023 LTR_FINDER Started

Wed Feb 1 01:33:11 +01 2023 LTR_HARVEST Started

Wed Feb 1 01:34:36 +01 2023 LTR_FINDER & LTR_HARVEST Done

Wed Feb 1 01:34:36 +01 2023 LTR_retriever Started

Wed Feb 1 03:40:30 +01 2023 LTR_retriever Done

Wed Feb 1 03:40:31 +01 2023 LTRdigest Started

Wed Feb 1 03:47:06 +01 2023 LTRdigest Done

Wed Feb 1 03:47:06 +01 2023 TEsorter Started

Wed Feb 1 03:49:06 +01 2023 TEsorter Done

Wed Feb 1 03:49:06 +01 2023 Filtering TEsorter results Started

Wed Feb 1 03:49:06 +01 2023 Mergeing of LTR_retriever, LTRdigest, and TEsorter results

Wed Feb 1 03:49:36 +01 2023 Calculation of the LTR-RT insertion time

Wed Feb 1 03:52:57 +01 2023 Preparing R plots files

Wed Feb 1 03:53:04 +01 2023 LTR-RT insertion time plots done

Wed Feb 1 03:53:05 +01 2023 LTR-RT-gene chimeras started

Wed Feb 1 03:53:21 +01 2023 LTR-RT-gene chimeras done

Wed Feb 1 03:53:21 +01 2023 LTR-RT near genes started

Wed Feb 1 03:53:47 +01 2023 LTR-RT near genes done

Wed Feb 1 03:53:47 +01 2023 Visualization of gene density and LTR-RTs across chromosomes

Wed Feb 1 03:54:38 +01 2023 MegaLTR Done, The results saved in (Brassica_rapa_10/Collected_Files)

12 CPU

#############################################

############## MegaLTR v2.0 ##############

#############################################

Contributors: Morad M Mokhtar, Achraf El Allali

Wed Feb 1 00:57:17 +01 2023 Start time

Parameters: -A 3 -F Brassica_rapa.fna -G Brassica_rapa.gff -T Brassica_rapa_trna.fa -P Brassica_rapa_12 -l 100 -L 7000 -d 1000 -D 15000 -S 85 -M 20 -B rexdb -C 20 -V 0.001 -Q 80-80-80 -E rexdb -R 0.000000015 -U 1000 -X 1000 -W 1000000 -N 9 -t 12

Check the FASTA File format.

Wed Feb 1 00:57:23 +01 2023 LTR_FINDER Started

Wed Feb 1 01:27:35 +01 2023 LTR_HARVEST Started

Wed Feb 1 01:28:48 +01 2023 LTR_FINDER & LTR_HARVEST Done

Wed Feb 1 01:28:48 +01 2023 LTR_retriever Started

Wed Feb 1 03:18:50 +01 2023 LTR_retriever Done

Wed Feb 1 03:18:52 +01 2023 LTRdigest Started

Wed Feb 1 03:25:26 +01 2023 LTRdigest Done

Wed Feb 1 03:25:26 +01 2023 TEsorter Started

Wed Feb 1 03:27:42 +01 2023 TEsorter Done

Wed Feb 1 03:27:42 +01 2023 Filtering TEsorter results Started

Wed Feb 1 03:27:42 +01 2023 Mergeing of LTR_retriever, LTRdigest, and TEsorter results

Wed Feb 1 03:28:05 +01 2023 Calculation of the LTR-RT insertion time

Wed Feb 1 03:31:26 +01 2023 Preparing R plots files

Wed Feb 1 03:31:33 +01 2023 LTR-RT insertion time plots done

Wed Feb 1 03:31:34 +01 2023 LTR-RT-gene chimeras started

Wed Feb 1 03:31:50 +01 2023 LTR-RT-gene chimeras done

Wed Feb 1 03:31:50 +01 2023 LTR-RT near genes started

Wed Feb 1 03:32:16 +01 2023 LTR-RT near genes done

Wed Feb 1 03:32:16 +01 2023 Visualization of gene density and LTR-RTs across chromosomes

Wed Feb 1 03:33:07 +01 2023 MegaLTR Done, The results saved in (Brassica_rapa_12/Collected_Files)

14 CPU

#############################################

############## MegaLTR v2.0 ##############

#############################################

Contributors: Morad M Mokhtar, Achraf El Allali

Wed Feb 1 00:57:26 +01 2023 Start time

Parameters: -A 3 -F Brassica_rapa.fna -G Brassica_rapa.gff -T Brassica_rapa_trna.fa -P Brassica_rapa_14 -l 100 -L 7000 -d 1000 -D 15000 -S 85 -M 20 -B rexdb -C 20 -V 0.001 -Q 80-80-80 -E rexdb -R 0.000000015 -U 1000 -X 1000 -W 1000000 -N 9 -t 14

Check the FASTA File format.

Wed Feb 1 00:57:32 +01 2023 LTR_FINDER Started

Wed Feb 1 01:23:31 +01 2023 LTR_HARVEST Started

Wed Feb 1 01:24:36 +01 2023 LTR_FINDER & LTR_HARVEST Done

Wed Feb 1 01:24:36 +01 2023 LTR_retriever Started

Wed Feb 1 03:04:29 +01 2023 LTR_retriever Done

Wed Feb 1 03:04:30 +01 2023 LTRdigest Started

Wed Feb 1 03:11:05 +01 2023 LTRdigest Done

Wed Feb 1 03:11:05 +01 2023 TEsorter Started

Wed Feb 1 03:12:38 +01 2023 TEsorter Done

Wed Feb 1 03:12:38 +01 2023 Filtering TEsorter results Started

Wed Feb 1 03:12:38 +01 2023 Mergeing of LTR_retriever, LTRdigest, and TEsorter results

Wed Feb 1 03:12:59 +01 2023 Calculation of the LTR-RT insertion time

Wed Feb 1 03:16:20 +01 2023 Preparing R plots files

Wed Feb 1 03:16:27 +01 2023 LTR-RT insertion time plots done

Wed Feb 1 03:16:28 +01 2023 LTR-RT-gene chimeras started

Wed Feb 1 03:16:45 +01 2023 LTR-RT-gene chimeras done

Wed Feb 1 03:16:45 +01 2023 LTR-RT near genes started

Wed Feb 1 03:17:12 +01 2023 LTR-RT near genes done

Wed Feb 1 03:17:12 +01 2023 Visualization of gene density and LTR-RTs across chromosomes

Wed Feb 1 03:18:01 +01 2023 MegaLTR Done, The results saved in (Brassica_rapa_14/Collected_Files)

16 CPU

#############################################

############## MegaLTR v2.0 ##############

#############################################

Contributors: Morad M Mokhtar, Achraf El Allali

Wed Feb 1 00:57:37 +01 2023 Start time

Parameters: -A 3 -F Brassica_rapa.fna -G Brassica_rapa.gff -T Brassica_rapa_trna.fa -P Brassica_rapa_16 -l 100 -L 7000 -d 1000 -D 15000 -S 85 -M 20 -B rexdb -C 20 -V 0.001 -Q 80-80-80 -E rexdb -R 0.000000015 -U 1000 -X 1000 -W 1000000 -N 9 -t 16

Check the FASTA File format.

Wed Feb 1 00:57:43 +01 2023 LTR_FINDER Started

Wed Feb 1 01:20:37 +01 2023 LTR_HARVEST Started

Wed Feb 1 01:21:37 +01 2023 LTR_FINDER & LTR_HARVEST Done

Wed Feb 1 01:21:37 +01 2023 LTR_retriever Started

Wed Feb 1 02:54:03 +01 2023 LTR_retriever Done

Wed Feb 1 02:54:04 +01 2023 LTRdigest Started

Wed Feb 1 03:00:47 +01 2023 LTRdigest Done

Wed Feb 1 03:00:47 +01 2023 TEsorter Started

Wed Feb 1 03:02:15 +01 2023 TEsorter Done

Wed Feb 1 03:02:15 +01 2023 Filtering TEsorter results Started

Wed Feb 1 03:02:15 +01 2023 Mergeing of LTR_retriever, LTRdigest, and TEsorter results

Wed Feb 1 03:02:36 +01 2023 Calculation of the LTR-RT insertion time

Wed Feb 1 03:05:57 +01 2023 Preparing R plots files

Wed Feb 1 03:06:03 +01 2023 LTR-RT insertion time plots done

Wed Feb 1 03:06:05 +01 2023 LTR-RT-gene chimeras started

Wed Feb 1 03:06:21 +01 2023 LTR-RT-gene chimeras done

Wed Feb 1 03:06:21 +01 2023 LTR-RT near genes started

Wed Feb 1 03:06:46 +01 2023 LTR-RT near genes done

Wed Feb 1 03:06:46 +01 2023 Visualization of gene density and LTR-RTs across chromosomes

Wed Feb 1 03:07:37 +01 2023 MegaLTR Done, The results saved in (Brassica_rapa_16/Collected_Files)

18 CPU

#############################################

############## MegaLTR v2.0 ##############

#############################################

Contributors: Morad M Mokhtar, Achraf El Allali

Wed Feb 1 00:57:45 +01 2023 Start time

Parameters: -A 3 -F Brassica_rapa.fna -G Brassica_rapa.gff -T Brassica_rapa_trna.fa -P Brassica_rapa_18 -l 100 -L 7000 -d 1000 -D 15000 -S 85 -M 20 -B rexdb -C 20 -V 0.001 -Q 80-80-80 -E rexdb -R 0.000000015 -U 1000 -X 1000 -W 1000000 -N 9 -t 18

Check the FASTA File format.

Wed Feb 1 00:57:51 +01 2023 LTR_FINDER Started

Wed Feb 1 01:18:21 +01 2023 LTR_HARVEST Started

Wed Feb 1 01:19:16 +01 2023 LTR_FINDER & LTR_HARVEST Done

Wed Feb 1 01:19:16 +01 2023 LTR_retriever Started

Wed Feb 1 02:47:30 +01 2023 LTR_retriever Done

Wed Feb 1 02:47:32 +01 2023 LTRdigest Started

Wed Feb 1 02:54:09 +01 2023 LTRdigest Done

Wed Feb 1 02:54:09 +01 2023 TEsorter Started

Wed Feb 1 02:55:49 +01 2023 TEsorter Done

Wed Feb 1 02:55:49 +01 2023 Filtering TEsorter results Started

Wed Feb 1 02:55:49 +01 2023 Mergeing of LTR_retriever, LTRdigest, and TEsorter results

Wed Feb 1 02:56:05 +01 2023 Calculation of the LTR-RT insertion time

Wed Feb 1 02:59:25 +01 2023 Preparing R plots files

Wed Feb 1 02:59:32 +01 2023 LTR-RT insertion time plots done

Wed Feb 1 02:59:33 +01 2023 LTR-RT-gene chimeras started

Wed Feb 1 02:59:50 +01 2023 LTR-RT-gene chimeras done

Wed Feb 1 02:59:50 +01 2023 LTR-RT near genes started

Wed Feb 1 03:00:15 +01 2023 LTR-RT near genes done

Wed Feb 1 03:00:15 +01 2023 Visualization of gene density and LTR-RTs across chromosomes

Wed Feb 1 03:01:07 +01 2023 MegaLTR Done, The results saved in (Brassica_rapa_18/Collected_Files)

20 CPU

#############################################

############## MegaLTR v2.0 ##############

#############################################

Contributors: Morad M Mokhtar, Achraf El Allali

Wed Feb 1 00:57:55 +01 2023 Start time

Parameters: -A 3 -F Brassica_rapa.fna -G Brassica_rapa.gff -T Brassica_rapa_trna.fa -P Brassica_rapa_20 -l 100 -L 7000 -d 1000 -D 15000 -S 85 -M 20 -B rexdb -C 20 -V 0.001 -Q 80-80-80 -E rexdb -R 0.000000015 -U 1000 -X 1000 -W 1000000 -N 9 -t 20

Check the FASTA File format.

Wed Feb 1 00:58:01 +01 2023 LTR_FINDER Started

Wed Feb 1 01:16:38 +01 2023 LTR_HARVEST Started

Wed Feb 1 01:17:29 +01 2023 LTR_FINDER & LTR_HARVEST Done

Wed Feb 1 01:17:29 +01 2023 LTR_retriever Started

Wed Feb 1 02:42:19 +01 2023 LTR_retriever Done

Wed Feb 1 02:42:20 +01 2023 LTRdigest Started

Wed Feb 1 02:48:54 +01 2023 LTRdigest Done

Wed Feb 1 02:48:54 +01 2023 TEsorter Started

Wed Feb 1 02:50:10 +01 2023 TEsorter Done

Wed Feb 1 02:50:10 +01 2023 Filtering TEsorter results Started

Wed Feb 1 02:50:10 +01 2023 Mergeing of LTR_retriever, LTRdigest, and TEsorter results

Wed Feb 1 02:50:26 +01 2023 Calculation of the LTR-RT insertion time

Wed Feb 1 02:53:47 +01 2023 Preparing R plots files

Wed Feb 1 02:53:53 +01 2023 LTR-RT insertion time plots done

Wed Feb 1 02:53:55 +01 2023 LTR-RT-gene chimeras started

Wed Feb 1 02:54:11 +01 2023 LTR-RT-gene chimeras done

Wed Feb 1 02:54:11 +01 2023 LTR-RT near genes started

Wed Feb 1 02:54:38 +01 2023 LTR-RT near genes done

Wed Feb 1 02:54:38 +01 2023 Visualization of gene density and LTR-RTs across chromosomes

Wed Feb 1 02:55:29 +01 2023 MegaLTR Done, The results saved in (Brassica_rapa_20/Collected_Files)

22 CPU

#############################################

############## MegaLTR v2.0 ##############

#############################################

Contributors: Morad M Mokhtar, Achraf El Allali

Wed Feb 1 00:58:04 +01 2023 Start time

Parameters: -A 3 -F Brassica_rapa.fna -G Brassica_rapa.gff -T Brassica_rapa_trna.fa -P Brassica_rapa_22 -l 100 -L 7000 -d 1000 -D 15000 -S 85 -M 20 -B rexdb -C 20 -V 0.001 -Q 80-80-80 -E rexdb -R 0.000000015 -U 1000 -X 1000 -W 1000000 -N 9 -t 22

Check the FASTA File format.

Wed Feb 1 00:58:10 +01 2023 LTR_FINDER Started

Wed Feb 1 01:15:14 +01 2023 LTR_HARVEST Started

Wed Feb 1 01:16:03 +01 2023 LTR_FINDER & LTR_HARVEST Done

Wed Feb 1 01:16:03 +01 2023 LTR_retriever Started

Wed Feb 1 02:38:24 +01 2023 LTR_retriever Done

Wed Feb 1 02:38:26 +01 2023 LTRdigest Started

Wed Feb 1 02:45:04 +01 2023 LTRdigest Done

Wed Feb 1 02:45:04 +01 2023 TEsorter Started

Wed Feb 1 02:46:16 +01 2023 TEsorter Done

Wed Feb 1 02:46:16 +01 2023 Filtering TEsorter results Started

Wed Feb 1 02:46:16 +01 2023 Mergeing of LTR_retriever, LTRdigest, and TEsorter results

Wed Feb 1 02:46:32 +01 2023 Calculation of the LTR-RT insertion time

Wed Feb 1 02:49:53 +01 2023 Preparing R plots files

Wed Feb 1 02:50:00 +01 2023 LTR-RT insertion time plots done

Wed Feb 1 02:50:01 +01 2023 LTR-RT-gene chimeras started

Wed Feb 1 02:50:17 +01 2023 LTR-RT-gene chimeras done

Wed Feb 1 02:50:17 +01 2023 LTR-RT near genes started

Wed Feb 1 02:50:43 +01 2023 LTR-RT near genes done

Wed Feb 1 02:50:43 +01 2023 Visualization of gene density and LTR-RTs across chromosomes

Wed Feb 1 02:51:34 +01 2023 MegaLTR Done, The results saved in (Brassica_rapa_22/Collected_Files)

24 CPU

#############################################

############## MegaLTR v2.0 ##############

#############################################

Contributors: Morad M Mokhtar, Achraf El Allali

Wed Feb 1 00:58:12 +01 2023 Start time

Parameters: -A 3 -F Brassica_rapa.fna -G Brassica_rapa.gff -T Brassica_rapa_trna.fa -P Brassica_rapa_24 -l 100 -L 7000 -d 1000 -D 15000 -S 85 -M 20 -B rexdb -C 20 -V 0.001 -Q 80-80-80 -E rexdb -R 0.000000015 -U 1000 -X 1000 -W 1000000 -N 9 -t 24

Check the FASTA File format.

Wed Feb 1 00:58:18 +01 2023 LTR_FINDER Started

Wed Feb 1 01:14:06 +01 2023 LTR_HARVEST Started

Wed Feb 1 01:14:52 +01 2023 LTR_FINDER & LTR_HARVEST Done

Wed Feb 1 01:14:52 +01 2023 LTR_retriever Started

Wed Feb 1 02:35:03 +01 2023 LTR_retriever Done

Wed Feb 1 02:35:04 +01 2023 LTRdigest Started

Wed Feb 1 02:41:38 +01 2023 LTRdigest Done

Wed Feb 1 02:41:38 +01 2023 TEsorter Started

Wed Feb 1 02:43:02 +01 2023 TEsorter Done

Wed Feb 1 02:43:02 +01 2023 Filtering TEsorter results Started

Wed Feb 1 02:43:02 +01 2023 Mergeing of LTR_retriever, LTRdigest, and TEsorter results

Wed Feb 1 02:43:18 +01 2023 Calculation of the LTR-RT insertion time

Wed Feb 1 02:46:38 +01 2023 Preparing R plots files

Wed Feb 1 02:46:44 +01 2023 LTR-RT insertion time plots done

Wed Feb 1 02:46:46 +01 2023 LTR-RT-gene chimeras started

Wed Feb 1 02:47:01 +01 2023 LTR-RT-gene chimeras done

Wed Feb 1 02:47:01 +01 2023 LTR-RT near genes started

Wed Feb 1 02:47:27 +01 2023 LTR-RT near genes done

Wed Feb 1 02:47:27 +01 2023 Visualization of gene density and LTR-RTs across chromosomes

Wed Feb 1 02:48:18 +01 2023 MegaLTR Done, The results saved in (Brassica_rapa_24/Collected_Files)

26 CPU

#############################################

############## MegaLTR v2.0 ##############

#############################################

Contributors: Morad M Mokhtar, Achraf El Allali

Wed Feb 1 00:58:20 +01 2023 Start time

Parameters: -A 3 -F Brassica_rapa.fna -G Brassica_rapa.gff -T Brassica_rapa_trna.fa -P Brassica_rapa_26 -l 100 -L 7000 -d 1000 -D 15000 -S 85 -M 20 -B rexdb -C 20 -V 0.001 -Q 80-80-80 -E rexdb -R 0.000000015 -U 1000 -X 1000 -W 1000000 -N 9 -t 26

Check the FASTA File format.

Wed Feb 1 00:58:26 +01 2023 LTR_FINDER Started

Wed Feb 1 01:13:09 +01 2023 LTR_HARVEST Started

Wed Feb 1 01:13:53 +01 2023 LTR_FINDER & LTR_HARVEST Done

Wed Feb 1 01:13:53 +01 2023 LTR_retriever Started

Wed Feb 1 02:31:37 +01 2023 LTR_retriever Done

Wed Feb 1 02:31:39 +01 2023 LTRdigest Started

Wed Feb 1 02:38:12 +01 2023 LTRdigest Done

Wed Feb 1 02:38:12 +01 2023 TEsorter Started

Wed Feb 1 02:39:21 +01 2023 TEsorter Done

Wed Feb 1 02:39:21 +01 2023 Filtering TEsorter results Started

Wed Feb 1 02:39:21 +01 2023 Mergeing of LTR_retriever, LTRdigest, and TEsorter results

Wed Feb 1 02:39:32 +01 2023 Calculation of the LTR-RT insertion time

Wed Feb 1 02:42:52 +01 2023 Preparing R plots files

Wed Feb 1 02:42:59 +01 2023 LTR-RT insertion time plots done

Wed Feb 1 02:43:00 +01 2023 LTR-RT-gene chimeras started

Wed Feb 1 02:43:16 +01 2023 LTR-RT-gene chimeras done

Wed Feb 1 02:43:16 +01 2023 LTR-RT near genes started

Wed Feb 1 02:43:42 +01 2023 LTR-RT near genes done

Wed Feb 1 02:43:42 +01 2023 Visualization of gene density and LTR-RTs across chromosomes

Wed Feb 1 02:44:33 +01 2023 MegaLTR Done, The results saved in (Brassica_rapa_26/Collected_Files)

28 CPU

#############################################

############## MegaLTR v2.0 ##############

#############################################

Contributors: Morad M Mokhtar, Achraf El Allali

Wed Feb 1 00:58:29 +01 2023 Start time

Parameters: -A 3 -F Brassica_rapa.fna -G Brassica_rapa.gff -T Brassica_rapa_trna.fa -P Brassica_rapa_28 -l 100 -L 7000 -d 1000 -D 15000 -S 85 -M 20 -B rexdb -C 20 -V 0.001 -Q 80-80-80 -E rexdb -R 0.000000015 -U 1000 -X 1000 -W 1000000 -N 9 -t 28

Check the FASTA File format.

Wed Feb 1 00:58:35 +01 2023 LTR_FINDER Started

Wed Feb 1 01:12:21 +01 2023 LTR_HARVEST Started

Wed Feb 1 01:13:03 +01 2023 LTR_FINDER & LTR_HARVEST Done

Wed Feb 1 01:13:03 +01 2023 LTR_retriever Started

Wed Feb 1 02:29:25 +01 2023 LTR_retriever Done

Wed Feb 1 02:29:26 +01 2023 LTRdigest Started

Wed Feb 1 02:36:03 +01 2023 LTRdigest Done

Wed Feb 1 02:36:03 +01 2023 TEsorter Started

Wed Feb 1 02:37:09 +01 2023 TEsorter Done

Wed Feb 1 02:37:09 +01 2023 Filtering TEsorter results Started

Wed Feb 1 02:37:09 +01 2023 Mergeing of LTR_retriever, LTRdigest, and TEsorter results

Wed Feb 1 02:37:20 +01 2023 Calculation of the LTR-RT insertion time

Wed Feb 1 02:40:40 +01 2023 Preparing R plots files

Wed Feb 1 02:40:47 +01 2023 LTR-RT insertion time plots done

Wed Feb 1 02:40:48 +01 2023 LTR-RT-gene chimeras started

Wed Feb 1 02:41:03 +01 2023 LTR-RT-gene chimeras done

Wed Feb 1 02:41:03 +01 2023 LTR-RT near genes started

Wed Feb 1 02:41:29 +01 2023 LTR-RT near genes done

Wed Feb 1 02:41:29 +01 2023 Visualization of gene density and LTR-RTs across chromosomes

Wed Feb 1 02:42:20 +01 2023 MegaLTR Done, The results saved in (Brassica_rapa_28/Collected_Files)

30 CPU

#############################################

############## MegaLTR v2.0 ##############

#############################################

Contributors: Morad M Mokhtar, Achraf El Allali

Wed Feb 1 00:58:39 +01 2023 Start time

Parameters: -A 3 -F Brassica_rapa.fna -G Brassica_rapa.gff -T Brassica_rapa_trna.fa -P Brassica_rapa_30 -l 100 -L 7000 -d 1000 -D 15000 -S 85 -M 20 -B rexdb -C 20 -V 0.001 -Q 80-80-80 -E rexdb -R 0.000000015 -U 1000 -X 1000 -W 1000000 -N 9 -t 30

Check the FASTA File format.

Wed Feb 1 00:58:46 +01 2023 LTR_FINDER Started

Wed Feb 1 01:11:45 +01 2023 LTR_HARVEST Started

Wed Feb 1 01:12:26 +01 2023 LTR_FINDER & LTR_HARVEST Done

Wed Feb 1 01:12:26 +01 2023 LTR_retriever Started

Wed Feb 1 02:27:29 +01 2023 LTR_retriever Done

Wed Feb 1 02:27:30 +01 2023 LTRdigest Started

Wed Feb 1 02:34:04 +01 2023 LTRdigest Done

Wed Feb 1 02:34:04 +01 2023 TEsorter Started

Wed Feb 1 02:35:20 +01 2023 TEsorter Done

Wed Feb 1 02:35:20 +01 2023 Filtering TEsorter results Started

Wed Feb 1 02:35:20 +01 2023 Mergeing of LTR_retriever, LTRdigest, and TEsorter results

Wed Feb 1 02:35:31 +01 2023 Calculation of the LTR-RT insertion time

Wed Feb 1 02:38:51 +01 2023 Preparing R plots files

Wed Feb 1 02:38:58 +01 2023 LTR-RT insertion time plots done

Wed Feb 1 02:38:59 +01 2023 LTR-RT-gene chimeras started

Wed Feb 1 02:39:15 +01 2023 LTR-RT-gene chimeras done

Wed Feb 1 02:39:15 +01 2023 LTR-RT near genes started

Wed Feb 1 02:39:41 +01 2023 LTR-RT near genes done

Wed Feb 1 02:39:41 +01 2023 Visualization of gene density and LTR-RTs across chromosomes

Wed Feb 1 02:40:35 +01 2023 MegaLTR Done, The results saved in (Brassica_rapa_30/Collected_Files)
