# Supplementary material for: MegaLTR: a web server and standalone pipeline for detecting and annotating LTR-retrotransposons in plant genomes
Source: Front Plant Sci. 2023 Sep 20;14:1237426. doi: 10.3389/fpls.2023.1237426 (PMC10552921; doi:10.3389/fpls.2023.1237426)
Supplement: Supplementary file 3 [file DataSheet_3.docx]

MegaLTR: A web server and standalone pipeline for detecting and annotating LTR-Retrotransposons in plant genomes

**Supplementary File 3**

**The times of the steps indicated by EDTA for the 26 genomes examined**

*Arabidopsis thaliana*

Mon Jan 23 19:08:54 +01 2023 EDTA_raw: Check dependencies, prepare working directories.

Mon Jan 23 19:08:54 +01 2023 Arabidopsis_thaliana.fna

Mon Jan 23 19:08:56 +01 2023 Start to find LTR candidates.

Mon Jan 23 19:08:56 +01 2023 Identify LTR retrotransposon candidates from scratch.

Mon Jan 23 19:22:39 +01 2023 Finish finding LTR candidates.

Mon Jan 23 19:22:39 +01 2023 Execution of EDTA_raw.pl is finished!

*Zea mays*

Mon Jan 23 19:49:52 +01 2023 EDTA_raw: Check dependencies, prepare working directories.

Mon Jan 23 19:49:52 +01 2023 Zea_mays.fna

Mon Jan 23 19:50:19 +01 2023 The longest sequence ID in the genome contains 14 characters, which is longer than the limit (13)

Trying to reformat seq IDs...

Attempt 1...

Mon Jan 23 19:50:32 +01 2023 Seq ID conversion successful!

Mon Jan 23 19:50:32 +01 2023 Start to find LTR candidates.

Mon Jan 23 19:50:32 +01 2023 Identify LTR retrotransposon candidates from scratch.

Tue Jan 24 23:29:27 +01 2023 Finish finding LTR candidates.

Tue Jan 24 23:29:27 +01 2023 Execution of EDTA_raw.pl is finished!

*Arabidopsis lyrata*

Mon Jan 23 19:49:52 +01 2023 EDTA_raw: Check dependencies, prepare working directories.

Mon Jan 23 19:49:52 +01 2023 Arabidopsis_lyrata.fna

Mon Jan 23 19:49:55 +01 2023 The longest sequence ID in the genome contains 14 characters, which is longer than the limit (13)

Trying to reformat seq IDs...

Attempt 1...

Mon Jan 23 19:49:57 +01 2023 Seq ID conversion successful!

Mon Jan 23 19:49:57 +01 2023 Start to find LTR candidates.

Mon Jan 23 19:49:57 +01 2023 Identify LTR retrotransposon candidates from scratch.

Mon Jan 23 20:04:16 +01 2023 Finish finding LTR candidates.

Mon Jan 23 20:04:16 +01 2023 Execution of EDTA_raw.pl is finished!

*Citrus clementina*

Mon Jan 23 19:50:41 +01 2023 EDTA_raw: Check dependencies, prepare working directories.

Mon Jan 23 19:50:41 +01 2023 Citrus_clementina.fna

Mon Jan 23 19:50:46 +01 2023 The longest sequence ID in the genome contains 14 characters, which is longer than the limit (13)

Trying to reformat seq IDs...

Attempt 1...

Mon Jan 23 19:50:48 +01 2023 Seq ID conversion successful!

Mon Jan 23 19:50:48 +01 2023 Start to find LTR candidates.

Mon Jan 23 19:50:48 +01 2023 Identify LTR retrotransposon candidates from scratch.

Mon Jan 23 20:14:28 +01 2023 Finish finding LTR candidates.

Mon Jan 23 20:14:28 +01 2023 Execution of EDTA_raw.pl is finished!

*Brassica rapa*

Mon Jan 23 19:50:41 +01 2023 EDTA_raw: Check dependencies, prepare working directories.

Mon Jan 23 19:50:41 +01 2023 Brassica_rapa.fna

Mon Jan 23 19:50:46 +01 2023 The longest sequence ID in the genome contains 14 characters, which is longer than the limit (13)

Trying to reformat seq IDs...

Attempt 1...

Mon Jan 23 19:50:48 +01 2023 Seq ID conversion successful!

Mon Jan 23 19:50:48 +01 2023 Start to find LTR candidates.

Mon Jan 23 19:50:48 +01 2023 Identify LTR retrotransposon candidates from scratch.

Mon Jan 23 20:28:15 +01 2023 Finish finding LTR candidates.

Mon Jan 23 20:28:15 +01 2023 Execution of EDTA_raw.pl is finished!

*Citrus unshiu*

Mon Jan 23 19:50:41 +01 2023 EDTA_raw: Check dependencies, prepare working directories.

Mon Jan 23 19:50:41 +01 2023 Citrus_unshiu.fna

Mon Jan 23 19:50:46 +01 2023 The longest sequence ID in the genome contains 14 characters, which is longer than the limit (13)

Trying to reformat seq IDs...

Attempt 1...

Mon Jan 23 19:50:49 +01 2023 Seq ID conversion successful!

Mon Jan 23 19:50:49 +01 2023 Start to find LTR candidates.

Mon Jan 23 19:50:49 +01 2023 Identify LTR retrotransposon candidates from scratch.

Mon Jan 23 20:10:34 +01 2023 Finish finding LTR candidates.

Mon Jan 23 20:10:34 +01 2023 Execution of EDTA_raw.pl is finished!

*Cucumis sativus*

Mon Jan 23 19:50:41 +01 2023 EDTA_raw: Check dependencies, prepare working directories.

Mon Jan 23 19:50:41 +01 2023 Cucumis_sativus.fna

Mon Jan 23 19:50:45 +01 2023 The longest sequence ID in the genome contains 14 characters, which is longer than the limit (13)

Trying to reformat seq IDs...

Attempt 1...

Mon Jan 23 19:50:46 +01 2023 Seq ID conversion successful!

Mon Jan 23 19:50:46 +01 2023 Start to find LTR candidates.

Mon Jan 23 19:50:46 +01 2023 Identify LTR retrotransposon candidates from scratch.

Mon Jan 23 19:56:54 +01 2023 Finish finding LTR candidates.

Mon Jan 23 19:56:54 +01 2023 Execution of EDTA_raw.pl is finished!

*Glycine max*

Mon Jan 23 19:50:41 +01 2023 EDTA_raw: Check dependencies, prepare working directories.

Mon Jan 23 19:50:41 +01 2023 Glycine_max.fna

Mon Jan 23 19:50:54 +01 2023 The longest sequence ID in the genome contains 14 characters, which is longer than the limit (13)

Trying to reformat seq IDs...

Attempt 1...

Mon Jan 23 19:51:00 +01 2023 Seq ID conversion successful!

Mon Jan 23 19:51:00 +01 2023 Start to find LTR candidates.

Mon Jan 23 19:51:00 +01 2023 Identify LTR retrotransposon candidates from scratch.

Mon Jan 23 21:03:04 +01 2023 Finish finding LTR candidates.

Mon Jan 23 21:03:04 +01 2023 Execution of EDTA_raw.pl is finished!

*Medicago truncatula*

Mon Jan 23 19:50:41 +01 2023 EDTA_raw: Check dependencies, prepare working directories.

Mon Jan 23 19:50:41 +01 2023 Medicago_truncatula.fna

Mon Jan 23 19:50:47 +01 2023 The longest sequence ID in the genome contains 14 characters, which is longer than the limit (13)

Trying to reformat seq IDs...

Attempt 1...

Mon Jan 23 19:50:50 +01 2023 Seq ID conversion successful!

Mon Jan 23 19:50:50 +01 2023 Start to find LTR candidates.

Mon Jan 23 19:50:50 +01 2023 Identify LTR retrotransposon candidates from scratch.

Mon Jan 23 20:21:44 +01 2023 Finish finding LTR candidates.

Mon Jan 23 20:21:44 +01 2023 Execution of EDTA_raw.pl is finished!

*Mikania micrantha*

Mon Jan 23 19:50:41 +01 2023 EDTA_raw: Check dependencies, prepare working directories.

Mon Jan 23 19:50:41 +01 2023 Mikania_micrantha.fna

Mon Jan 23 19:51:03 +01 2023 The longest sequence ID in the genome contains 14 characters, which is longer than the limit (13)

Trying to reformat seq IDs...

Attempt 1...

Mon Jan 23 19:51:14 +01 2023 Seq ID conversion successful!

Mon Jan 23 19:51:14 +01 2023 Start to find LTR candidates.

Mon Jan 23 19:51:14 +01 2023 Identify LTR retrotransposon candidates from scratch.

Tue Jan 24 10:33:38 +01 2023 Finish finding LTR candidates.

Tue Jan 24 10:33:38 +01 2023 Execution of EDTA_raw.pl is finished!

*Vitis vinifera*

Mon Jan 23 20:27:30 +01 2023 EDTA_raw: Check dependencies, prepare working directories.

Mon Jan 23 20:27:30 +01 2023 Vitis_vinifera.fna

Mon Jan 23 20:27:37 +01 2023 The longest sequence ID in the genome contains 14 characters, which is longer than the limit (13)

Trying to reformat seq IDs...

Attempt 1...

Mon Jan 23 20:27:40 +01 2023 Seq ID conversion successful!

Mon Jan 23 20:27:40 +01 2023 Start to find LTR candidates.

Mon Jan 23 20:27:40 +01 2023 Identify LTR retrotransposon candidates from scratch.

Mon Jan 23 21:02:05 +01 2023 Finish finding LTR candidates.

Mon Jan 23 21:02:05 +01 2023 Execution of EDTA_raw.pl is finished!

*Oryza sativa Japonica*

Mon Jan 23 19:50:40 +01 2023 EDTA_raw: Check dependencies, prepare working directories.

Mon Jan 23 19:50:40 +01 2023 Oryza_sativa_Japonica.fna

Mon Jan 23 19:50:46 +01 2023 The longest sequence ID in the genome contains 14 characters, which is longer than the limit (13)

Trying to reformat seq IDs...

Attempt 1...

Mon Jan 23 19:50:48 +01 2023 Seq ID conversion successful!

Mon Jan 23 19:50:48 +01 2023 Start to find LTR candidates.

Mon Jan 23 19:50:48 +01 2023 Identify LTR retrotransposon candidates from scratch.

Mon Jan 23 20:15:36 +01 2023 Finish finding LTR candidates.

Mon Jan 23 20:15:36 +01 2023 Execution of EDTA_raw.pl is finished!

*Panicum hallii*

Mon Jan 23 19:50:40 +01 2023 EDTA_raw: Check dependencies, prepare working directories.

Mon Jan 23 19:50:40 +01 2023 Panicum_hallii.fna

Mon Jan 23 19:50:48 +01 2023 The longest sequence ID in the genome contains 14 characters, which is longer than the limit (13)

Trying to reformat seq IDs...

Attempt 1...

Mon Jan 23 19:50:51 +01 2023 Seq ID conversion successful!

Mon Jan 23 19:50:51 +01 2023 Start to find LTR candidates.

Mon Jan 23 19:50:51 +01 2023 Identify LTR retrotransposon candidates from scratch.

Mon Jan 23 21:13:45 +01 2023 Finish finding LTR candidates.

Mon Jan 23 21:13:45 +01 2023 Execution of EDTA_raw.pl is finished!

*Phoenix dactylifera*

Mon Jan 23 19:50:40 +01 2023 EDTA_raw: Check dependencies, prepare working directories.

Mon Jan 23 19:50:40 +01 2023 Phoenix_dactylifera.fna

Mon Jan 23 19:50:50 +01 2023 The longest sequence ID in the genome contains 14 characters, which is longer than the limit (13)

Trying to reformat seq IDs...

Attempt 1...

Mon Jan 23 19:50:55 +01 2023 Seq ID conversion successful!

Mon Jan 23 19:50:55 +01 2023 Start to find LTR candidates.

Mon Jan 23 19:50:55 +01 2023 Identify LTR retrotransposon candidates from scratch.

Mon Jan 23 22:19:38 +01 2023 Finish finding LTR candidates.

Mon Jan 23 22:19:38 +01 2023 Execution of EDTA_raw.pl is finished!

*Physcomitrella patens*

Mon Jan 23 19:50:41 +01 2023 EDTA_raw: Check dependencies, prepare working directories.

Mon Jan 23 19:50:41 +01 2023 Physcomitrella_patens.fna

Mon Jan 23 19:50:48 +01 2023 The longest sequence ID in the genome contains 14 characters, which is longer than the limit (13)

Trying to reformat seq IDs...

Attempt 1...

Mon Jan 23 19:50:51 +01 2023 Seq ID conversion successful!

Mon Jan 23 19:50:51 +01 2023 Start to find LTR candidates.

Mon Jan 23 19:50:51 +01 2023 Identify LTR retrotransposon candidates from scratch.

Mon Jan 23 20:17:01 +01 2023 Finish finding LTR candidates.

Mon Jan 23 20:17:01 +01 2023 Execution of EDTA_raw.pl is finished!

*Populus trichocarpa*

Mon Jan 23 19:54:10 +01 2023 EDTA_raw: Check dependencies, prepare working directories.

Mon Jan 23 19:54:10 +01 2023 Populus_trichocarpa.fna

Mon Jan 23 19:54:17 +01 2023 The longest sequence ID in the genome contains 14 characters, which is longer than the limit (13)

Trying to reformat seq IDs...

Attempt 1...

Mon Jan 23 19:54:19 +01 2023 Seq ID conversion successful!

Mon Jan 23 19:54:19 +01 2023 Start to find LTR candidates.

Mon Jan 23 19:54:19 +01 2023 Identify LTR retrotransposon candidates from scratch.

Mon Jan 23 20:20:47 +01 2023 Finish finding LTR candidates.

Mon Jan 23 20:20:47 +01 2023 Execution of EDTA_raw.pl is finished!

*Prunus persica*

Mon Jan 23 19:57:03 +01 2023 EDTA_raw: Check dependencies, prepare working directories.

Mon Jan 23 19:57:03 +01 2023 Prunus_persica.fna

Mon Jan 23 19:57:07 +01 2023 The longest sequence ID in the genome contains 14 characters, which is longer than the limit (13)

Trying to reformat seq IDs...

Attempt 1...

Mon Jan 23 19:57:08 +01 2023 Seq ID conversion successful!

Mon Jan 23 19:57:08 +01 2023 Start to find LTR candidates.

Mon Jan 23 19:57:08 +01 2023 Identify LTR retrotransposon candidates from scratch.

Mon Jan 23 20:27:20 +01 2023 Finish finding LTR candidates.

Mon Jan 23 20:27:20 +01 2023 Execution of EDTA_raw.pl is finished!

*Rosa chinensis*

Mon Jan 23 20:02:08 +01 2023 EDTA_raw: Check dependencies, prepare working directories.

Mon Jan 23 20:02:08 +01 2023 Rosa_chinensis.fna

Mon Jan 23 20:02:15 +01 2023 The longest sequence ID in the genome contains 14 characters, which is longer than the limit (13)

Trying to reformat seq IDs...

Attempt 1...

Mon Jan 23 20:02:18 +01 2023 Seq ID conversion successful!

Mon Jan 23 20:02:18 +01 2023 Start to find LTR candidates.

Mon Jan 23 20:02:18 +01 2023 Identify LTR retrotransposon candidates from scratch.

Mon Jan 23 21:45:40 +01 2023 Finish finding LTR candidates.

Mon Jan 23 21:45:40 +01 2023 Execution of EDTA_raw.pl is finished!

*Salvia splendens*

Mon Jan 23 20:04:24 +01 2023 EDTA_raw: Check dependencies, prepare working directories.

Mon Jan 23 20:04:24 +01 2023 Salvia_splendens.fna

Mon Jan 23 20:04:35 +01 2023 The longest sequence ID in the genome contains 14 characters, which is longer than the limit (13)

Trying to reformat seq IDs...

Attempt 1...

Mon Jan 23 20:04:40 +01 2023 Seq ID conversion successful!

Mon Jan 23 20:04:40 +01 2023 Start to find LTR candidates.

Mon Jan 23 20:04:40 +01 2023 Identify LTR retrotransposon candidates from scratch.

Mon Jan 23 23:22:56 +01 2023 Finish finding LTR candidates.

Mon Jan 23 23:22:56 +01 2023 Execution of EDTA_raw.pl is finished!

*Selaginella moellendorffii*

Mon Jan 23 20:10:42 +01 2023 EDTA_raw: Check dependencies, prepare working directories.

Mon Jan 23 20:10:42 +01 2023 Selaginella_moellendorffii.fna

Mon Jan 23 20:10:46 +01 2023 The longest sequence ID in the genome contains 14 characters, which is longer than the limit (13)

Trying to reformat seq IDs...

Attempt 1...

Mon Jan 23 20:10:47 +01 2023 Seq ID conversion successful!

Mon Jan 23 20:10:47 +01 2023 Start to find LTR candidates.

Mon Jan 23 20:10:47 +01 2023 Identify LTR retrotransposon candidates from scratch.

Mon Jan 23 20:20:22 +01 2023 Finish finding LTR candidates.

Mon Jan 23 20:20:22 +01 2023 Execution of EDTA_raw.pl is finished!

*Sesamum indicum*

Mon Jan 23 20:14:37 +01 2023 EDTA_raw: Check dependencies, prepare working directories.

Mon Jan 23 20:14:37 +01 2023 Sesamum_indicum.fna

Mon Jan 23 20:14:41 +01 2023 The longest sequence ID in the genome contains 14 characters, which is longer than the limit (13)

Trying to reformat seq IDs...

Attempt 1...

Mon Jan 23 20:14:43 +01 2023 Seq ID conversion successful!

Mon Jan 23 20:14:43 +01 2023 Start to find LTR candidates.

Mon Jan 23 20:14:43 +01 2023 Identify LTR retrotransposon candidates from scratch.

Mon Jan 23 20:42:43 +01 2023 Finish finding LTR candidates.

Mon Jan 23 20:42:43 +01 2023 Execution of EDTA_raw.pl is finished!

*Setaria viridis*

Mon Jan 23 20:15:45 +01 2023 EDTA_raw: Check dependencies, prepare working directories.

Mon Jan 23 20:15:45 +01 2023 Setaria_viridis.fna

Mon Jan 23 20:15:51 +01 2023 The longest sequence ID in the genome contains 14 characters, which is longer than the limit (13)

Trying to reformat seq IDs...

Attempt 1...

Mon Jan 23 20:15:53 +01 2023 Seq ID conversion successful!

Mon Jan 23 20:15:53 +01 2023 Start to find LTR candidates.

Mon Jan 23 20:15:53 +01 2023 Identify LTR retrotransposon candidates from scratch.

Mon Jan 23 20:51:25 +01 2023 Finish finding LTR candidates.

Mon Jan 23 20:51:25 +01 2023 Execution of EDTA_raw.pl is finished!

*Solanum lycopersicum*

Mon Jan 23 20:17:10 +01 2023 EDTA_raw: Check dependencies, prepare working directories.

Mon Jan 23 20:17:10 +01 2023 Solanum_lycopersicum.fna

Mon Jan 23 20:17:21 +01 2023 The longest sequence ID in the genome contains 14 characters, which is longer than the limit (13)

Trying to reformat seq IDs...

Attempt 1...

Mon Jan 23 20:17:26 +01 2023 Seq ID conversion successful!

Mon Jan 23 20:17:26 +01 2023 Start to find LTR candidates.

Mon Jan 23 20:17:26 +01 2023 Identify LTR retrotransposon candidates from scratch.

Mon Jan 23 20:52:46 +01 2023 Finish finding LTR candidates.

Mon Jan 23 20:52:46 +01 2023 Execution of EDTA_raw.pl is finished!

*Solanum pennellii*

Mon Jan 23 20:17:48 +01 2023 EDTA_raw: Check dependencies, prepare working directories.

Mon Jan 23 20:17:48 +01 2023 Solanum_pennellii.fna

Mon Jan 23 20:18:00 +01 2023 Start to find LTR candidates.

Mon Jan 23 20:18:00 +01 2023 Identify LTR retrotransposon candidates from scratch.

Mon Jan 23 20:59:31 +01 2023 Finish finding LTR candidates.

Mon Jan 23 20:59:31 +01 2023 Execution of EDTA_raw.pl is finished!

*Sorghum bicolor*

Mon Jan 23 20:20:30 +01 2023 EDTA_raw: Check dependencies, prepare working directories.

Mon Jan 23 20:20:30 +01 2023 Sorghum_bicolor.fna

Mon Jan 23 20:20:40 +01 2023 The longest sequence ID in the genome contains 14 characters, which is longer than the limit (13)

Trying to reformat seq IDs...

Attempt 1...

Mon Jan 23 20:20:44 +01 2023 Seq ID conversion successful!

Mon Jan 23 20:20:44 +01 2023 Start to find LTR candidates.

Mon Jan 23 20:20:44 +01 2023 Identify LTR retrotransposon candidates from scratch.

Mon Jan 23 22:55:18 +01 2023 Finish finding LTR candidates.

Mon Jan 23 22:55:18 +01 2023 Execution of EDTA_raw.pl is finished!

*Trifolium pratense*

Mon Jan 23 20:20:55 +01 2023 EDTA_raw: Check dependencies, prepare working directories.

Mon Jan 23 20:20:55 +01 2023 Trifolium_pratense.fna

Mon Jan 23 20:21:02 +01 2023 The longest sequence ID in the genome contains 14 characters, which is longer than the limit (13)

Trying to reformat seq IDs...

Attempt 1...

Mon Jan 23 20:21:04 +01 2023 Seq ID conversion successful!

Mon Jan 23 20:21:04 +01 2023 Start to find LTR candidates.

Mon Jan 23 20:21:04 +01 2023 Identify LTR retrotransposon candidates from scratch.

Mon Jan 23 21:20:41 +01 2023 Finish finding LTR candidates.

Mon Jan 23 21:20:41 +01 2023 Execution of EDTA_raw.pl is finished!
