# Supplementary material for: MegaLTR: a web server and standalone pipeline for detecting and annotating LTR-retrotransposons in plant genomes
Source: Front Plant Sci. 2023 Sep 20;14:1237426. doi: 10.3389/fpls.2023.1237426 (PMC10552921; doi:10.3389/fpls.2023.1237426)
Supplement: Supplementary file 4 [file DataSheet_4.docx]

MegaLTR: A web server and standalone pipeline for detecting and annotating LTR-Retrotransposons in plant genomes

**Supplementary File 4**

**The times of the steps indicated by MegaLTR for the 26 genomes examined**

*Arabidopsis lyrata*

#############################################

############## MegaLTR v2.0 ##############

#############################################

Contributors: Morad M Mokhtar, Achraf El Allali

Parameters: -A 3 -F /Arabidopsis_lyrata/Arabidopsis_lyrata.fna -G /Arabidopsis_lyrata/Arabidopsis_lyrata.gff -T Arabidopsis_lyrata_trna.fa -P Arabidopsis_lyrata -l 100 -L 7000 -d 1000 -D 15000 -S 85 -M 20 -B rexdb -C 20 -V 0.001 -Q 80-80-80 -E rexdb -R 0.000000015 -U 1000 -X 1000 -W 1000000 -N 9 -t 56

Check the FASTA File format.

Sun Jan 29 01:09:01 +01 2023 Start time

Sun Jan 29 01:09:08 +01 2023 LTR_FINDER & LTR_HARVEST Started

Sun Jan 29 01:13:00 +01 2023 LTR_FINDER & LTR_HARVEST Done

Sun Jan 29 01:13:01 +01 2023 LTRdigest Started

Sun Jan 29 01:15:30 +01 2023 LTRdigest Done

Sun Jan 29 01:15:30 +01 2023 TEsorter Started

Sun Jan 29 01:16:47 +01 2023 TEsorter Done

Sun Jan 29 01:16:47 +01 2023 Filtering TEsorter results Started

Sun Jan 29 01:16:47 +01 2023 Mergeing of LTR_retriever, LTRdigest, and TEsorter results

Sun Jan 29 01:17:05 +01 2023 Calculation of the LTR-RT insertion time

Sun Jan 29 01:18:03 +01 2023 Preparing R plots files

Sun Jan 29 01:18:10 +01 2023 LTR-RT insertion time plots done

Sun Jan 29 01:18:10 +01 2023 LTR-RT-gene chimeras started

Sun Jan 29 01:18:15 +01 2023 LTR-RT-gene chimeras done

Sun Jan 29 01:18:15 +01 2023 LTR-RT near genes started

Sun Jan 29 01:18:23 +01 2023 LTR-RT near genes done

Sun Jan 29 01:18:23 +01 2023 Visualization of gene density and LTR-RTs across chromosomes

Sun Jan 29 01:19:10 +01 2023 MegaLTR Done, The results saved in (Results/Arabidopsis_lyrata/Collected_Files)

*Arabidopsis thaliana*

#############################################

############## MegaLTR v2.0 ##############

#############################################

Contributors: Morad M Mokhtar, Achraf El Allali

Sun Jan 29 00:46:11 +01 2023 Start time

Parameters: -A 3 -F /Arabidopsis_thaliana/Arabidopsis_thaliana.fna -G /Arabidopsis_thaliana/Arabidopsis_thaliana.gff -T Arabidopsis_thaliana_trna.fa -P Arabidopsis_thaliana -l 100 -L 7000 -d 1000 -D 15000 -S 85 -M 20 -B rexdb -C 20 -V 0.001 -Q 80-80-80 -E rexdb -R 0.000000015 -U 1000 -X 1000 -W 1000000 -N 9 -t 56

Check the FASTA File format.

Sun Jan 29 00:46:17 +01 2023 LTR_FINDER & LTR_HARVEST Started

Sun Jan 29 00:53:08 +01 2023 LTR_FINDER & LTR_HARVEST Done

Sun Jan 29 00:53:09 +01 2023 LTRdigest Started

Sun Jan 29 00:54:03 +01 2023 LTRdigest Done

Sun Jan 29 00:54:03 +01 2023 TEsorter Started

Sun Jan 29 00:54:12 +01 2023 TEsorter Done

Sun Jan 29 00:54:12 +01 2023 Filtering TEsorter results Started

Sun Jan 29 00:54:12 +01 2023 Mergeing of LTR_retriever, LTRdigest, and TEsorter results

Sun Jan 29 00:54:15 +01 2023 Calculation of the LTR-RT insertion time

Sun Jan 29 00:54:24 +01 2023 Preparing R plots files

Sun Jan 29 00:54:30 +01 2023 LTR-RT insertion time plots done

Sun Jan 29 00:54:30 +01 2023 LTR-RT-gene chimeras started

Sun Jan 29 00:54:32 +01 2023 LTR-RT-gene chimeras done

Sun Jan 29 00:54:32 +01 2023 LTR-RT near genes started

Sun Jan 29 00:54:34 +01 2023 LTR-RT near genes done

Sun Jan 29 00:54:34 +01 2023 Visualization of gene density and LTR-RTs across chromosomes

Sun Jan 29 00:55:10 +01 2023 MegaLTR Done, The results saved in (Results/Arabidopsis_thaliana/Collected_Files)

*Brassica rapa*

#############################################

############## MegaLTR v2.0 ##############

#############################################

Contributors: Morad M Mokhtar, Achraf El Allali

Parameters: -A 3 -F /Brassica_rapa/Brassica_rapa.fna -G /Brassica_rapa/Brassica_rapa.gff -T Brassica_rapa_trna.fa -P Brassica_rapa -l 100 -L 7000 -d 1000 -D 15000 -S 85 -M 20 -B rexdb -C 20 -V 0.001 -Q 80-80-80 -E rexdb -R 0.000000015 -U 1000 -X 1000 -W 1000000 -N 9 -t 56

Check the FASTA File format.

Sun Jan 29 01:29:40 +01 2023 Start time

Sun Jan 29 01:29:50 +01 2023 LTR_FINDER & LTR_HARVEST Started

Sun Jan 29 02:02:09 +01 2023 LTR_FINDER & LTR_HARVEST Done

Sun Jan 29 02:02:10 +01 2023 LTRdigest Started

Sun Jan 29 02:09:10 +01 2023 LTRdigest Done

Sun Jan 29 02:09:10 +01 2023 TEsorter Started

Sun Jan 29 02:11:21 +01 2023 TEsorter Done

Sun Jan 29 02:11:21 +01 2023 Filtering TEsorter results Started

Sun Jan 29 02:11:21 +01 2023 Mergeing of LTR_retriever, LTRdigest, and TEsorter results

Sun Jan 29 02:11:52 +01 2023 Calculation of the LTR-RT insertion time

Sun Jan 29 02:15:22 +01 2023 Preparing R plots files

Sun Jan 29 02:15:28 +01 2023 LTR-RT insertion time plots done

Sun Jan 29 02:15:28 +01 2023 LTR-RT-gene chimeras started

Sun Jan 29 02:15:45 +01 2023 LTR-RT-gene chimeras done

Sun Jan 29 02:15:45 +01 2023 LTR-RT near genes started

Sun Jan 29 02:16:13 +01 2023 LTR-RT near genes done

Sun Jan 29 02:16:13 +01 2023 Visualization of gene density and LTR-RTs across chromosomes

Sun Jan 29 02:17:05 +01 2023 MegaLTR Done, The results saved in (Results/Brassica_rapa/Collected_Files)

*Citrus clementina*

#############################################

############## MegaLTR v2.0 ##############

#############################################

Contributors: Morad M Mokhtar, Achraf El Allali

Parameters: -A 3 -F /Citrus_clementina/Citrus_clementina.fna -G /Citrus_clementina/Citrus_clementina.gff -T Citrus_clementina_trna.fa -P Citrus_clementina -l 100 -L 7000 -d 1000 -D 15000 -S 85 -M 20 -B rexdb -C 20 -V 0.001 -Q 80-80-80 -E rexdb -R 0.000000015 -U 1000 -X 1000 -W 1000000 -N 9 -t 56

Check the FASTA File format.

Sun Jan 29 02:17:06 +01 2023 Start time

Sun Jan 29 02:17:15 +01 2023 LTR_FINDER & LTR_HARVEST Started

Sun Jan 29 02:24:01 +01 2023 LTR_FINDER & LTR_HARVEST Done

Sun Jan 29 02:24:02 +01 2023 LTRdigest Started

Sun Jan 29 02:27:55 +01 2023 LTRdigest Done

Sun Jan 29 02:27:55 +01 2023 TEsorter Started

Sun Jan 29 02:29:29 +01 2023 TEsorter Done

Sun Jan 29 02:29:29 +01 2023 Filtering TEsorter results Started

Sun Jan 29 02:29:29 +01 2023 Mergeing of LTR_retriever, LTRdigest, and TEsorter results

Sun Jan 29 02:29:57 +01 2023 Calculation of the LTR-RT insertion time

Sun Jan 29 02:31:40 +01 2023 Preparing R plots files

Sun Jan 29 02:31:46 +01 2023 LTR-RT insertion time plots done

Sun Jan 29 02:31:46 +01 2023 LTR-RT-gene chimeras started

Sun Jan 29 02:31:52 +01 2023 LTR-RT-gene chimeras done

Sun Jan 29 02:31:52 +01 2023 LTR-RT near genes started

Sun Jan 29 02:32:00 +01 2023 LTR-RT near genes done

Sun Jan 29 02:32:00 +01 2023 Visualization of gene density and LTR-RTs across chromosomes

Sun Jan 29 02:32:54 +01 2023 MegaLTR Done, The results saved in (Results/Citrus_clementina/Collected_Files)

*Citrus unshiu*

#############################################

############## MegaLTR v2.0 ##############

#############################################

Contributors: Morad M Mokhtar, Achraf El Allali

Parameters: -A 3 -F /Citrus_unshiu/Citrus_unshiu.fna -G /Citrus_unshiu/Citrus_unshiu.gff -T Citrus_unshiu_trna.fa -P Citrus_unshiu -l 100 -L 7000 -d 1000 -D 15000 -S 85 -M 20 -B rexdb -C 20 -V 0.001 -Q 80-80-80 -E rexdb -R 0.000000015 -U 1000 -X 1000 -W 1000000 -N 9 -t 56

Check the FASTA File format.

Sun Jan 29 02:32:54 +01 2023 Start time

Sun Jan 29 02:33:03 +01 2023 LTR_FINDER & LTR_HARVEST Started

Sun Jan 29 02:41:55 +01 2023 LTR_FINDER & LTR_HARVEST Done

Sun Jan 29 02:41:56 +01 2023 LTRdigest Started

Sun Jan 29 02:47:14 +01 2023 LTRdigest Done

Sun Jan 29 02:47:14 +01 2023 TEsorter Started

Sun Jan 29 02:47:42 +01 2023 TEsorter Done

Sun Jan 29 02:47:42 +01 2023 Filtering TEsorter results Started

Sun Jan 29 02:47:42 +01 2023 Mergeing of LTR_retriever, LTRdigest, and TEsorter results

Sun Jan 29 02:48:12 +01 2023 Calculation of the LTR-RT insertion time

Sun Jan 29 02:48:35 +01 2023 Preparing R plots files

Sun Jan 29 02:48:42 +01 2023 LTR-RT insertion time plots done

Sun Jan 29 02:48:42 +01 2023 LTR-RT-gene chimeras started

Sun Jan 29 02:48:44 +01 2023 LTR-RT-gene chimeras done

Sun Jan 29 02:48:44 +01 2023 LTR-RT near genes started

Sun Jan 29 02:48:48 +01 2023 LTR-RT near genes done

Sun Jan 29 02:48:48 +01 2023 Visualization of gene density and LTR-RTs across chromosomes

Sun Jan 29 02:49:22 +01 2023 MegaLTR Done, The results saved in (Results/Citrus_unshiu/Collected_Files)

*Cucumis sativus*

#############################################

############## MegaLTR v2.0 ##############

#############################################

Contributors: Morad M Mokhtar, Achraf El Allali

Parameters: -A 3 -F /Cucumis_sativus/Cucumis_sativus.fna -G /Cucumis_sativus/Cucumis_sativus.gff -T Cucumis_sativus_trna.fa -P Cucumis_sativus -l 100 -L 7000 -d 1000 -D 15000 -S 85 -M 20 -B rexdb -C 20 -V 0.001 -Q 80-80-80 -E rexdb -R 0.000000015 -U 1000 -X 1000 -W 1000000 -N 9 -t 56

Check the FASTA File format.

Sun Jan 29 03:11:50 +01 2023 Start time

Sun Jan 29 03:11:57 +01 2023 LTR_FINDER & LTR_HARVEST Started

Sun Jan 29 03:16:04 +01 2023 LTR_FINDER & LTR_HARVEST Done

Sun Jan 29 03:16:05 +01 2023 LTRdigest Started

Sun Jan 29 03:18:24 +01 2023 LTRdigest Done

Sun Jan 29 03:18:24 +01 2023 TEsorter Started

Sun Jan 29 03:18:46 +01 2023 TEsorter Done

Sun Jan 29 03:18:46 +01 2023 Filtering TEsorter results Started

Sun Jan 29 03:18:46 +01 2023 Mergeing of LTR_retriever, LTRdigest, and TEsorter results

Sun Jan 29 03:19:06 +01 2023 Calculation of the LTR-RT insertion time

Sun Jan 29 03:19:30 +01 2023 Preparing R plots files

Sun Jan 29 03:19:36 +01 2023 LTR-RT insertion time plots done

Sun Jan 29 03:19:36 +01 2023 LTR-RT-gene chimeras started

Sun Jan 29 03:19:37 +01 2023 LTR-RT-gene chimeras done

Sun Jan 29 03:19:37 +01 2023 LTR-RT near genes started

Sun Jan 29 03:19:39 +01 2023 LTR-RT near genes done

Sun Jan 29 03:19:39 +01 2023 Visualization of gene density and LTR-RTs across chromosomes

Sun Jan 29 03:20:17 +01 2023 MegaLTR Done, The results saved in (Results/Cucumis_sativus/Collected_Files)

*Glycine max*

#############################################

############## MegaLTR v2.0 ##############

#############################################

Contributors: Morad M Mokhtar, Achraf El Allali

Parameters: -A 3 -F /Glycine_max/Glycine_max.fna -G /Glycine_max/Glycine_max.gff -T Glycine_max_trna.fa -P Glycine_max -l 100 -L 7000 -d 1000 -D 15000 -S 85 -M 20 -B rexdb -C 20 -V 0.001 -Q 80-80-80 -E rexdb -R 0.000000015 -U 1000 -X 1000 -W 1000000 -N 9 -t 56

Check the FASTA File format.

Sun Jan 29 01:09:15 +01 2023 Start time

Sun Jan 29 01:09:40 +01 2023 LTR_FINDER & LTR_HARVEST Started

Sun Jan 29 01:31:35 +01 2023 LTR_FINDER & LTR_HARVEST Done

Sun Jan 29 01:31:37 +01 2023 LTRdigest Started

Sun Jan 29 01:47:51 +01 2023 LTRdigest Done

Sun Jan 29 01:47:51 +01 2023 TEsorter Started

Sun Jan 29 01:52:02 +01 2023 TEsorter Done

Sun Jan 29 01:52:02 +01 2023 Filtering TEsorter results Started

Sun Jan 29 01:52:02 +01 2023 Mergeing of LTR_retriever, LTRdigest, and TEsorter results

Sun Jan 29 01:53:30 +01 2023 Calculation of the LTR-RT insertion time

Sun Jan 29 01:59:53 +01 2023 Preparing R plots files

Sun Jan 29 02:00:00 +01 2023 LTR-RT insertion time plots done

Sun Jan 29 02:00:00 +01 2023 LTR-RT-gene chimeras started

Sun Jan 29 02:00:39 +01 2023 LTR-RT-gene chimeras done

Sun Jan 29 02:00:39 +01 2023 LTR-RT near genes started

Sun Jan 29 02:01:38 +01 2023 LTR-RT near genes done

Sun Jan 29 02:01:38 +01 2023 Visualization of gene density and LTR-RTs across chromosomes

Sun Jan 29 02:03:07 +01 2023 MegaLTR Done, The results saved in (Results/Glycine_max/Collected_Files)

*Medicago truncatula*

#############################################

############## MegaLTR v2.0 ##############

#############################################

Contributors: Morad M Mokhtar, Achraf El Allali

Parameters: -A 3 -F /Medicago_truncatula/Medicago_truncatula.fna -G /Medicago_truncatula/Medicago_truncatula.gff -T Medicago_truncatula_trna.fa -P Medicago_truncatula -l 100 -L 7000 -d 1000 -D 15000 -S 85 -M 20 -B rexdb -C 20 -V 0.001 -Q 80-80-80 -E rexdb -R 0.000000015 -U 1000 -X 1000 -W 1000000 -N 9 -t 56

Check the FASTA File format.

Sun Jan 29 02:03:07 +01 2023 Start time

Sun Jan 29 02:03:20 +01 2023 LTR_FINDER & LTR_HARVEST Started

Sun Jan 29 02:10:22 +01 2023 LTR_FINDER & LTR_HARVEST Done

Sun Jan 29 02:10:23 +01 2023 LTRdigest Started

Sun Jan 29 02:16:24 +01 2023 LTRdigest Done

Sun Jan 29 02:16:24 +01 2023 TEsorter Started

Sun Jan 29 02:19:10 +01 2023 TEsorter Done

Sun Jan 29 02:19:10 +01 2023 Filtering TEsorter results Started

Sun Jan 29 02:19:10 +01 2023 Mergeing of LTR_retriever, LTRdigest, and TEsorter results

Sun Jan 29 02:19:49 +01 2023 Calculation of the LTR-RT insertion time

Sun Jan 29 02:27:35 +01 2023 Preparing R plots files

Sun Jan 29 02:27:42 +01 2023 LTR-RT insertion time plots done

Sun Jan 29 02:27:42 +01 2023 LTR-RT-gene chimeras started

Sun Jan 29 02:27:53 +01 2023 LTR-RT-gene chimeras done

Sun Jan 29 02:27:53 +01 2023 LTR-RT near genes started

Sun Jan 29 02:28:11 +01 2023 LTR-RT near genes done

Sun Jan 29 02:28:11 +01 2023 Visualization of gene density and LTR-RTs across chromosomes

Sun Jan 29 02:29:08 +01 2023 MegaLTR Done, The results saved in (Results/Medicago_truncatula/Collected_Files)

*Mikania micrantha*

#############################################

############## MegaLTR v2.0 ##############

#############################################

Contributors: Morad M Mokhtar, Achraf El Allali

Parameters: -A 3 -F /Mikania_micrantha/Mikania_micrantha.fna -G /Mikania_micrantha/Mikania_micrantha.gff -T Mikania_micrantha_trna.fa -P Mikania_micrantha -l 100 -L 7000 -d 1000 -D 15000 -S 85 -M 20 -B rexdb -C 20 -V 0.001 -Q 80-80-80 -E rexdb -R 0.000000015 -U 1000 -X 1000 -W 1000000 -N 9 -t 56

Check the FASTA File format.

Sun Jan 29 02:29:08 +01 2023 Start time

Sun Jan 29 02:30:03 +01 2023 LTR_FINDER & LTR_HARVEST Started

Sun Jan 29 03:51:32 +01 2023 LTR_FINDER & LTR_HARVEST Done

Sun Jan 29 03:51:33 +01 2023 LTRdigest Started

Sun Jan 29 04:42:56 +01 2023 LTRdigest Done

Sun Jan 29 04:42:56 +01 2023 TEsorter Started

Sun Jan 29 05:09:44 +01 2023 TEsorter Done

Sun Jan 29 05:09:44 +01 2023 Filtering TEsorter results Started

Sun Jan 29 05:09:44 +01 2023 Mergeing of LTR_retriever, LTRdigest, and TEsorter results

Sun Jan 29 05:13:04 +01 2023 Calculation of the LTR-RT insertion time

Sun Jan 29 07:07:49 +01 2023 Preparing R plots files

Sun Jan 29 07:07:56 +01 2023 LTR-RT insertion time plots done

Sun Jan 29 07:07:56 +01 2023 LTR-RT-gene chimeras started

Sun Jan 29 07:13:43 +01 2023 LTR-RT-gene chimeras done

Sun Jan 29 07:13:43 +01 2023 LTR-RT near genes started

Sun Jan 29 07:17:03 +01 2023 LTR-RT near genes done

Sun Jan 29 07:17:03 +01 2023 Visualization of gene density and LTR-RTs across chromosomes

Sun Jan 29 07:20:33 +01 2023 MegaLTR Done, The results saved in (Results/Mikania_micrantha/Collected_Files)

*Oryza sativa Japonica*

#############################################

############## MegaLTR v2.0 ##############

#############################################

Contributors: Morad M Mokhtar, Achraf El Allali

Parameters: -A 3 -F /Oryza_sativa_Japonica/Oryza_sativa_Japonica.fna -G /Oryza_sativa_Japonica/Oryza_sativa_Japonica.gff -T Oryza_sativa_Japonica_trna.fa -P Oryza_sativa_Japonica -l 100 -L 7000 -d 1000 -D 15000 -S 85 -M 20 -B rexdb -C 20 -V 0.001 -Q 80-80-80 -E rexdb -R 0.000000013 -U 1000 -X 1000 -W 1000000 -N 9 -t 56

Check the FASTA File format.

Sun Jan 29 07:20:33 +01 2023 Start time

Sun Jan 29 07:20:45 +01 2023 LTR_FINDER & LTR_HARVEST Started

Sun Jan 29 07:33:45 +01 2023 LTR_FINDER & LTR_HARVEST Done

Sun Jan 29 07:33:46 +01 2023 LTRdigest Started

Sun Jan 29 07:38:28 +01 2023 LTRdigest Done

Sun Jan 29 07:38:28 +01 2023 TEsorter Started

Sun Jan 29 07:40:50 +01 2023 TEsorter Done

Sun Jan 29 07:40:50 +01 2023 Filtering TEsorter results Started

Sun Jan 29 07:40:50 +01 2023 Mergeing of LTR_retriever, LTRdigest, and TEsorter results

Sun Jan 29 07:41:23 +01 2023 Calculation of the LTR-RT insertion time

Sun Jan 29 07:47:14 +01 2023 Preparing R plots files

Sun Jan 29 07:47:20 +01 2023 LTR-RT insertion time plots done

Sun Jan 29 07:47:20 +01 2023 LTR-RT-gene chimeras started

Sun Jan 29 07:47:29 +01 2023 LTR-RT-gene chimeras done

Sun Jan 29 07:47:29 +01 2023 LTR-RT near genes started

Sun Jan 29 07:47:42 +01 2023 LTR-RT near genes done

Sun Jan 29 07:47:42 +01 2023 Visualization of gene density and LTR-RTs across chromosomes

Sun Jan 29 07:48:40 +01 2023 MegaLTR Done, The results saved in (Results/Oryza_sativa_Japonica/Collected_Files)

*Panicum hallii*

#############################################

############## MegaLTR v2.0 ##############

#############################################

Contributors: Morad M Mokhtar, Achraf El Allali

Parameters: -A 3 -F /Panicum_hallii/Panicum_hallii.fna -G /Panicum_hallii/Panicum_hallii.gff -T Panicum_hallii_trna.fa -P Panicum_hallii -l 100 -L 7000 -d 1000 -D 15000 -S 85 -M 20 -B rexdb -C 20 -V 0.001 -Q 80-80-80 -E rexdb -R 0.000000013 -U 1000 -X 1000 -W 1000000 -N 9 -t 56

Check the FASTA File format.

Sun Jan 29 07:48:40 +01 2023 Start time

Sun Jan 29 07:48:56 +01 2023 LTR_FINDER & LTR_HARVEST Started

Sun Jan 29 08:09:07 +01 2023 LTR_FINDER & LTR_HARVEST Done

Sun Jan 29 08:09:08 +01 2023 LTRdigest Started

Sun Jan 29 08:17:16 +01 2023 LTRdigest Done

Sun Jan 29 08:17:16 +01 2023 TEsorter Started

Sun Jan 29 08:24:47 +01 2023 TEsorter Done

Sun Jan 29 08:24:47 +01 2023 Filtering TEsorter results Started

Sun Jan 29 08:24:48 +01 2023 Mergeing of LTR_retriever, LTRdigest, and TEsorter results

Sun Jan 29 08:25:37 +01 2023 Calculation of the LTR-RT insertion time

Sun Jan 29 08:49:47 +01 2023 Preparing R plots files

Sun Jan 29 08:49:54 +01 2023 LTR-RT insertion time plots done

Sun Jan 29 08:49:54 +01 2023 LTR-RT-gene chimeras started

Sun Jan 29 08:50:16 +01 2023 LTR-RT-gene chimeras done

Sun Jan 29 08:50:16 +01 2023 LTR-RT near genes started

Sun Jan 29 08:50:45 +01 2023 LTR-RT near genes done

Sun Jan 29 08:50:45 +01 2023 Visualization of gene density and LTR-RTs across chromosomes

Sun Jan 29 08:51:54 +01 2023 MegaLTR Done, The results saved in (Results/Panicum_hallii/Collected_Files)

*Phoenix dactylifera*

#############################################

############## MegaLTR v2.0 ##############

#############################################

Contributors: Morad M Mokhtar, Achraf El Allali

Parameters: -A 3 -F /Phoenix_dactylifera/Phoenix_dactylifera.fna -G /Phoenix_dactylifera/Phoenix_dactylifera.gff -T Phoenix_dactylifera_trna.fa -P Phoenix_dactylifera -l 100 -L 7000 -d 1000 -D 15000 -S 85 -M 20 -B rexdb -C 20 -V 0.001 -Q 80-80-80 -E rexdb -R 0.000000015 -U 1000 -X 1000 -W 1000000 -N 9 -t 56

Check the FASTA File format.

Sun Jan 29 08:51:55 +01 2023 Start time

Sun Jan 29 08:52:12 +01 2023 LTR_FINDER & LTR_HARVEST Started

Sun Jan 29 09:14:08 +01 2023 LTR_FINDER & LTR_HARVEST Done

Sun Jan 29 09:14:09 +01 2023 LTRdigest Started

Sun Jan 29 09:29:06 +01 2023 LTRdigest Done

Sun Jan 29 09:29:06 +01 2023 TEsorter Started

Sun Jan 29 09:40:20 +01 2023 TEsorter Done

Sun Jan 29 09:40:20 +01 2023 Filtering TEsorter results Started

Sun Jan 29 09:40:20 +01 2023 Mergeing of LTR_retriever, LTRdigest, and TEsorter results

Sun Jan 29 09:41:28 +01 2023 Calculation of the LTR-RT insertion time

Sun Jan 29 10:09:16 +01 2023 Preparing R plots files

Sun Jan 29 10:09:23 +01 2023 LTR-RT insertion time plots done

Sun Jan 29 10:09:23 +01 2023 LTR-RT-gene chimeras started

Sun Jan 29 10:10:23 +01 2023 LTR-RT-gene chimeras done

Sun Jan 29 10:10:23 +01 2023 LTR-RT near genes started

Sun Jan 29 10:11:27 +01 2023 LTR-RT near genes done

Sun Jan 29 10:11:27 +01 2023 Visualization of gene density and LTR-RTs across chromosomes

Sun Jan 29 10:12:34 +01 2023 MegaLTR Done, The results saved in (Results/Phoenix_dactylifera/Collected_Files)

*Physcomitrella patens*

#############################################

############## MegaLTR v2.0 ##############

#############################################

Contributors: Morad M Mokhtar, Achraf El Allali

Parameters: -A 3 -F /Physcomitrella_patens/Physcomitrella_patens.fna -G /Physcomitrella_patens/Physcomitrella_patens.gff -T Physcomitrella_patens_trna.fa -P Physcomitrella_patens -l 100 -L 7000 -d 1000 -D 15000 -S 85 -M 20 -B rexdb -C 20 -V 0.001 -Q 80-80-80 -E rexdb -R 0.000000015 -U 1000 -X 1000 -W 1000000 -N 9 -t 56

Check the FASTA File format.

Sun Jan 29 01:09:28 +01 2023 Start time

Sun Jan 29 01:09:40 +01 2023 LTR_FINDER & LTR_HARVEST Started

Sun Jan 29 01:16:20 +01 2023 LTR_FINDER & LTR_HARVEST Done

Sun Jan 29 01:16:21 +01 2023 LTRdigest Started

Sun Jan 29 01:22:41 +01 2023 LTRdigest Done

Sun Jan 29 01:22:41 +01 2023 TEsorter Started

Sun Jan 29 01:25:38 +01 2023 TEsorter Done

Sun Jan 29 01:25:38 +01 2023 Filtering TEsorter results Started

Sun Jan 29 01:25:38 +01 2023 Mergeing of LTR_retriever, LTRdigest, and TEsorter results

Sun Jan 29 01:26:18 +01 2023 Calculation of the LTR-RT insertion time

Sun Jan 29 01:28:29 +01 2023 Preparing R plots files

Sun Jan 29 01:28:35 +01 2023 LTR-RT insertion time plots done

Sun Jan 29 01:28:35 +01 2023 LTR-RT-gene chimeras started

Sun Jan 29 01:28:47 +01 2023 LTR-RT-gene chimeras done

Sun Jan 29 01:28:47 +01 2023 LTR-RT near genes started

Sun Jan 29 01:29:03 +01 2023 LTR-RT near genes done

Sun Jan 29 01:29:03 +01 2023 Visualization of gene density and LTR-RTs across chromosomes

Sun Jan 29 01:30:11 +01 2023 MegaLTR Done, The results saved in (Results/Physcomitrella_patens/Collected_Files)

*Populus trichocarpa*

#############################################

############## MegaLTR v2.0 ##############

#############################################

Contributors: Morad M Mokhtar, Achraf El Allali

Parameters: -A 3 -F /Populus_trichocarpa/Populus_trichocarpa.fna -G /Populus_trichocarpa/Populus_trichocarpa.gff -T Populus_trichocarpa_trna.fa -P Populus_trichocarpa -l 100 -L 7000 -d 1000 -D 15000 -S 85 -M 20 -B rexdb -C 20 -V 0.001 -Q 80-80-80 -E rexdb -R 0.000000015 -U 1000 -X 1000 -W 1000000 -N 9 -t 56

Check the FASTA File format.

Sun Jan 29 01:30:12 +01 2023 Start time

Sun Jan 29 01:30:23 +01 2023 LTR_FINDER & LTR_HARVEST Started

Sun Jan 29 01:39:52 +01 2023 LTR_FINDER & LTR_HARVEST Done

Sun Jan 29 01:39:53 +01 2023 LTRdigest Started

Sun Jan 29 01:44:57 +01 2023 LTRdigest Done

Sun Jan 29 01:44:57 +01 2023 TEsorter Started

Sun Jan 29 01:45:46 +01 2023 TEsorter Done

Sun Jan 29 01:45:46 +01 2023 Filtering TEsorter results Started

Sun Jan 29 01:45:46 +01 2023 Mergeing of LTR_retriever, LTRdigest, and TEsorter results

Sun Jan 29 01:46:22 +01 2023 Calculation of the LTR-RT insertion time

Sun Jan 29 01:47:37 +01 2023 Preparing R plots files

Sun Jan 29 01:47:44 +01 2023 LTR-RT insertion time plots done

Sun Jan 29 01:47:44 +01 2023 LTR-RT-gene chimeras started

Sun Jan 29 01:47:48 +01 2023 LTR-RT-gene chimeras done

Sun Jan 29 01:47:48 +01 2023 LTR-RT near genes started

Sun Jan 29 01:47:53 +01 2023 LTR-RT near genes done

Sun Jan 29 01:47:53 +01 2023 Visualization of gene density and LTR-RTs across chromosomes

Sun Jan 29 01:48:37 +01 2023 MegaLTR Done, The results saved in (Results/Populus_trichocarpa/Collected_Files)

*Prunus persica*

#############################################

############## MegaLTR v2.0 ##############

#############################################

Contributors: Morad M Mokhtar, Achraf El Allali

Parameters: -A 3 -F /Prunus_persica/Prunus_persica.fna -G /Prunus_persica/Prunus_persica.gff -T Prunus_persica_trna.fa -P Prunus_persica -l 100 -L 7000 -d 1000 -D 15000 -S 85 -M 20 -B rexdb -C 20 -V 0.001 -Q 80-80-80 -E rexdb -R 0.000000015 -U 1000 -X 1000 -W 1000000 -N 9 -t 56

Check the FASTA File format.

Sun Jan 29 01:48:37 +01 2023 Start time

Sun Jan 29 01:48:44 +01 2023 LTR_FINDER & LTR_HARVEST Started

Sun Jan 29 01:58:20 +01 2023 LTR_FINDER & LTR_HARVEST Done

Sun Jan 29 01:58:22 +01 2023 LTRdigest Started

Sun Jan 29 02:01:06 +01 2023 LTRdigest Done

Sun Jan 29 02:01:06 +01 2023 TEsorter Started

Sun Jan 29 02:02:10 +01 2023 TEsorter Done

Sun Jan 29 02:02:10 +01 2023 Filtering TEsorter results Started

Sun Jan 29 02:02:10 +01 2023 Mergeing of LTR_retriever, LTRdigest, and TEsorter results

Sun Jan 29 02:02:31 +01 2023 Calculation of the LTR-RT insertion time

Sun Jan 29 02:03:16 +01 2023 Preparing R plots files

Sun Jan 29 02:03:22 +01 2023 LTR-RT insertion time plots done

Sun Jan 29 02:03:22 +01 2023 LTR-RT-gene chimeras started

Sun Jan 29 02:03:25 +01 2023 LTR-RT-gene chimeras done

Sun Jan 29 02:03:25 +01 2023 LTR-RT near genes started

Sun Jan 29 02:03:30 +01 2023 LTR-RT near genes done

Sun Jan 29 02:03:30 +01 2023 Visualization of gene density and LTR-RTs across chromosomes

Sun Jan 29 02:04:17 +01 2023 MegaLTR Done, The results saved in (Results/Prunus_persica/Collected_Files)

*Rosa chinensis*

#############################################

############## MegaLTR v2.0 ##############

#############################################

Contributors: Morad M Mokhtar, Achraf El Allali

Parameters: -A 3 -F /Rosa_chinensis/Rosa_chinensis.fna -G /Rosa_chinensis/Rosa_chinensis.gff -T Rosa_chinensis_trna.fa -P Rosa_chinensis -l 100 -L 7000 -d 1000 -D 15000 -S 85 -M 20 -B rexdb -C 20 -V 0.001 -Q 80-80-80 -E rexdb -R 0.000000015 -U 1000 -X 1000 -W 1000000 -N 9 -t 56

Check the FASTA File format.

Sun Jan 29 02:36:04 +01 2023 Start time

Sun Jan 29 02:36:19 +01 2023 LTR_FINDER & LTR_HARVEST Started

Sun Jan 29 02:53:03 +01 2023 LTR_FINDER & LTR_HARVEST Done

Sun Jan 29 02:53:04 +01 2023 LTRdigest Started

Sun Jan 29 03:02:26 +01 2023 LTRdigest Done

Sun Jan 29 03:02:26 +01 2023 TEsorter Started

Sun Jan 29 03:09:09 +01 2023 TEsorter Done

Sun Jan 29 03:09:09 +01 2023 Filtering TEsorter results Started

Sun Jan 29 03:09:09 +01 2023 Mergeing of LTR_retriever, LTRdigest, and TEsorter results

Sun Jan 29 03:09:58 +01 2023 Calculation of the LTR-RT insertion time

Sun Jan 29 03:16:54 +01 2023 Preparing R plots files

Sun Jan 29 03:17:00 +01 2023 LTR-RT insertion time plots done

Sun Jan 29 03:17:00 +01 2023 LTR-RT-gene chimeras started

Sun Jan 29 03:17:46 +01 2023 LTR-RT-gene chimeras done

Sun Jan 29 03:17:46 +01 2023 LTR-RT near genes started

Sun Jan 29 03:18:52 +01 2023 LTR-RT near genes done

Sun Jan 29 03:18:52 +01 2023 Visualization of gene density and LTR-RTs across chromosomes

Sun Jan 29 03:20:14 +01 2023 MegaLTR Done, The results saved in (Results/Rosa_chinensis/Collected_Files)

*Salvia splendens*

#############################################

############## MegaLTR v2.0 ##############

#############################################

Contributors: Morad M Mokhtar, Achraf El Allali

Parameters: -A 3 -F /Salvia_splendens/Salvia_splendens.fna -G /Salvia_splendens/Salvia_splendens.gff -T Salvia_splendens_trna.fa -P Salvia_splendens -l 100 -L 7000 -d 1000 -D 15000 -S 85 -M 20 -B rexdb -C 20 -V 0.001 -Q 80-80-80 -E rexdb -R 0.000000015 -U 1000 -X 1000 -W 1000000 -N 9 -t 56

Check the FASTA File format.

Sun Jan 29 03:20:15 +01 2023 Start time

Sun Jan 29 03:20:37 +01 2023 LTR_FINDER & LTR_HARVEST Started

Sun Jan 29 04:39:46 +01 2023 LTR_FINDER & LTR_HARVEST Done

Sun Jan 29 04:39:47 +01 2023 LTRdigest Started

Sun Jan 29 05:05:40 +01 2023 LTRdigest Done

Sun Jan 29 05:05:40 +01 2023 TEsorter Started

Sun Jan 29 05:15:33 +01 2023 TEsorter Done

Sun Jan 29 05:15:33 +01 2023 Filtering TEsorter results Started

Sun Jan 29 05:15:33 +01 2023 Mergeing of LTR_retriever, LTRdigest, and TEsorter results

Sun Jan 29 05:16:55 +01 2023 Calculation of the LTR-RT insertion time

Sun Jan 29 05:30:22 +01 2023 Preparing R plots files

Sun Jan 29 05:30:29 +01 2023 LTR-RT insertion time plots done

Sun Jan 29 05:30:29 +01 2023 LTR-RT-gene chimeras started

Sun Jan 29 05:32:21 +01 2023 LTR-RT-gene chimeras done

Sun Jan 29 05:32:21 +01 2023 LTR-RT near genes started

Sun Jan 29 05:34:51 +01 2023 LTR-RT near genes done

Sun Jan 29 05:34:51 +01 2023 Visualization of gene density and LTR-RTs across chromosomes

Sun Jan 29 05:36:49 +01 2023 MegaLTR Done, The results saved in (Results/Salvia_splendens/Collected_Files)

*Selaginella moellendorffii*

#############################################

############## MegaLTR v2.0 ##############

#############################################

Contributors: Morad M Mokhtar, Achraf El Allali

Parameters: -A 3 -F /Selaginella_moellendorffii/Selaginella_moellendorffii.fna -G /Selaginella_moellendorffii/Selaginella_moellendorffii.gff -T Selaginella_moellendorffii_trna.fa -P Selaginella_moellendorffii -l 100 -L 7000 -d 1000 -D 15000 -S 85 -M 20 -B rexdb -C 20 -V 0.001 -Q 80-80-80 -E rexdb -R 0.000000015 -U 1000 -X 1000 -W 1000000 -N 9 -t 56

Check the FASTA File format.

Sun Jan 29 01:09:42 +01 2023 Start time

Sun Jan 29 01:09:48 +01 2023 LTR_FINDER & LTR_HARVEST Started

Sun Jan 29 01:14:01 +01 2023 LTR_FINDER & LTR_HARVEST Done

Sun Jan 29 01:14:02 +01 2023 LTRdigest Started

Sun Jan 29 01:17:14 +01 2023 LTRdigest Done

Sun Jan 29 01:17:14 +01 2023 TEsorter Started

Sun Jan 29 01:18:00 +01 2023 TEsorter Done

Sun Jan 29 01:18:00 +01 2023 Filtering TEsorter results Started

Sun Jan 29 01:18:00 +01 2023 Mergeing of LTR_retriever, LTRdigest, and TEsorter results

Sun Jan 29 01:18:18 +01 2023 Calculation of the LTR-RT insertion time

Sun Jan 29 01:19:22 +01 2023 Preparing R plots files

Sun Jan 29 01:19:28 +01 2023 LTR-RT insertion time plots done

Sun Jan 29 01:19:28 +01 2023 LTR-RT-gene chimeras started

Sun Jan 29 01:19:32 +01 2023 LTR-RT-gene chimeras done

Sun Jan 29 01:19:32 +01 2023 LTR-RT near genes started

Sun Jan 29 01:19:37 +01 2023 LTR-RT near genes done

Sun Jan 29 01:19:37 +01 2023 Visualization of gene density and LTR-RTs across chromosomes

Sun Jan 29 01:20:12 +01 2023 MegaLTR Done, The results saved in (Results/Selaginella_moellendorffii/Collected_Files)

*Sesamum indicum*

#############################################

############## MegaLTR v2.0 ##############

#############################################

Contributors: Morad M Mokhtar, Achraf El Allali

Parameters: -A 3 -F /Sesamum_indicum/Sesamum_indicum.fna -G /Sesamum_indicum/Sesamum_indicum.gff -T Sesamum_indicum_trna.fa -P Sesamum_indicum -l 100 -L 7000 -d 1000 -D 15000 -S 85 -M 20 -B rexdb -C 20 -V 0.001 -Q 80-80-80 -E rexdb -R 0.000000015 -U 1000 -X 1000 -W 1000000 -N 9 -t 56

Check the FASTA File format.

Sun Jan 29 01:20:12 +01 2023 Start time

Sun Jan 29 01:20:21 +01 2023 LTR_FINDER & LTR_HARVEST Started

Sun Jan 29 01:35:19 +01 2023 LTR_FINDER & LTR_HARVEST Done

Sun Jan 29 01:35:20 +01 2023 LTRdigest Started

Sun Jan 29 01:39:41 +01 2023 LTRdigest Done

Sun Jan 29 01:39:41 +01 2023 TEsorter Started

Sun Jan 29 01:40:06 +01 2023 TEsorter Done

Sun Jan 29 01:40:06 +01 2023 Filtering TEsorter results Started

Sun Jan 29 01:40:06 +01 2023 Mergeing of LTR_retriever, LTRdigest, and TEsorter results

Sun Jan 29 01:40:32 +01 2023 Calculation of the LTR-RT insertion time

Sun Jan 29 01:40:48 +01 2023 Preparing R plots files

Sun Jan 29 01:40:54 +01 2023 LTR-RT insertion time plots done

Sun Jan 29 01:40:54 +01 2023 LTR-RT-gene chimeras started

Sun Jan 29 01:40:56 +01 2023 LTR-RT-gene chimeras done

Sun Jan 29 01:40:56 +01 2023 LTR-RT near genes started

Sun Jan 29 01:40:58 +01 2023 LTR-RT near genes done

Sun Jan 29 01:40:58 +01 2023 Visualization of gene density and LTR-RTs across chromosomes

Sun Jan 29 01:41:35 +01 2023 MegaLTR Done, The results saved in (Results/Sesamum_indicum/Collected_Files)

*Setaria viridis*

#############################################

############## MegaLTR v2.0 ##############

#############################################

Contributors: Morad M Mokhtar, Achraf El Allali

Parameters: -A 3 -F /Setaria_viridis/Setaria_viridis.fna -G /Setaria_viridis/Setaria_viridis.gff -T Setaria_viridis_trna.fa -P Setaria_viridis -l 100 -L 7000 -d 1000 -D 15000 -S 85 -M 20 -B rexdb -C 20 -V 0.001 -Q 80-80-80 -E rexdb -R 0.000000013 -U 1000 -X 1000 -W 1000000 -N 9 -t 56

Check the FASTA File format.

Sun Jan 29 01:41:36 +01 2023 Start time

Sun Jan 29 01:41:46 +01 2023 LTR_FINDER & LTR_HARVEST Started

Sun Jan 29 01:53:49 +01 2023 LTR_FINDER & LTR_HARVEST Done

Sun Jan 29 01:53:50 +01 2023 LTRdigest Started

Sun Jan 29 01:58:00 +01 2023 LTRdigest Done

Sun Jan 29 01:58:00 +01 2023 TEsorter Started

Sun Jan 29 02:00:13 +01 2023 TEsorter Done

Sun Jan 29 02:00:13 +01 2023 Filtering TEsorter results Started

Sun Jan 29 02:00:13 +01 2023 Mergeing of LTR_retriever, LTRdigest, and TEsorter results

Sun Jan 29 02:00:48 +01 2023 Calculation of the LTR-RT insertion time

Sun Jan 29 02:04:56 +01 2023 Preparing R plots files

Sun Jan 29 02:05:02 +01 2023 LTR-RT insertion time plots done

Sun Jan 29 02:05:02 +01 2023 LTR-RT-gene chimeras started

Sun Jan 29 02:05:10 +01 2023 LTR-RT-gene chimeras done

Sun Jan 29 02:05:10 +01 2023 LTR-RT near genes started

Sun Jan 29 02:05:22 +01 2023 LTR-RT near genes done

Sun Jan 29 02:05:22 +01 2023 Visualization of gene density and LTR-RTs across chromosomes

Sun Jan 29 02:06:15 +01 2023 MegaLTR Done, The results saved in (Results/Setaria_viridis/Collected_Files)

*Solanum lycopersicum*

#############################################

############## MegaLTR v2.0 ##############

#############################################

Contributors: Morad M Mokhtar, Achraf El Allali

Parameters: -A 3 -F /Solanum_lycopersicum/Solanum_lycopersicum.fna -G /Solanum_lycopersicum/Solanum_lycopersicum.gff -T Solanum_lycopersicum_trna.fa -P Solanum_lycopersicum -l 100 -L 7000 -d 1000 -D 15000 -S 85 -M 20 -B rexdb -C 20 -V 0.001 -Q 80-80-80 -E rexdb -R 0.000000015 -U 1000 -X 1000 -W 1000000 -N 9 -t 56

Check the FASTA File format.

Sun Jan 29 02:06:16 +01 2023 Start time

Sun Jan 29 02:06:41 +01 2023 LTR_FINDER & LTR_HARVEST Started

Sun Jan 29 02:21:32 +01 2023 LTR_FINDER & LTR_HARVEST Done

Sun Jan 29 02:21:33 +01 2023 LTRdigest Started

Sun Jan 29 02:31:39 +01 2023 LTRdigest Done

Sun Jan 29 02:31:39 +01 2023 TEsorter Started

Sun Jan 29 02:33:15 +01 2023 TEsorter Done

Sun Jan 29 02:33:15 +01 2023 Filtering TEsorter results Started

Sun Jan 29 02:33:15 +01 2023 Mergeing of LTR_retriever, LTRdigest, and TEsorter results

Sun Jan 29 02:34:28 +01 2023 Calculation of the LTR-RT insertion time

Sun Jan 29 02:37:38 +01 2023 Preparing R plots files

Sun Jan 29 02:37:44 +01 2023 LTR-RT insertion time plots done

Sun Jan 29 02:37:44 +01 2023 LTR-RT-gene chimeras started

Sun Jan 29 02:37:50 +01 2023 LTR-RT-gene chimeras done

Sun Jan 29 02:37:50 +01 2023 LTR-RT near genes started

Sun Jan 29 02:38:00 +01 2023 LTR-RT near genes done

Sun Jan 29 02:38:00 +01 2023 Visualization of gene density and LTR-RTs across chromosomes

Sun Jan 29 02:38:56 +01 2023 MegaLTR Done, The results saved in (Results/Solanum_lycopersicum/Collected_Files)

*Solanum pennellii*

#############################################

############## MegaLTR v2.0 ##############

#############################################

Contributors: Morad M Mokhtar, Achraf El Allali

Parameters: -A 3 -F /Solanum_pennellii/Solanum_pennellii.fna -G /Solanum_pennellii/Solanum_pennellii.gff -T Solanum_pennellii_trna.fa -P Solanum_pennellii -l 100 -L 7000 -d 1000 -D 15000 -S 85 -M 20 -B rexdb -C 20 -V 0.001 -Q 80-80-80 -E rexdb -R 0.000000015 -U 1000 -X 1000 -W 1000000 -N 9 -t 56

Check the FASTA File format.

Sun Jan 29 02:38:56 +01 2023 Start time

Sun Jan 29 02:39:28 +01 2023 LTR_FINDER & LTR_HARVEST Started

Sun Jan 29 02:57:55 +01 2023 LTR_FINDER & LTR_HARVEST Done

Sun Jan 29 02:57:56 +01 2023 LTRdigest Started

Sun Jan 29 03:08:26 +01 2023 LTRdigest Done

Sun Jan 29 03:08:26 +01 2023 TEsorter Started

Sun Jan 29 03:09:57 +01 2023 TEsorter Done

Sun Jan 29 03:09:57 +01 2023 Filtering TEsorter results Started

Sun Jan 29 03:09:57 +01 2023 Mergeing of LTR_retriever, LTRdigest, and TEsorter results

Sun Jan 29 03:11:19 +01 2023 Calculation of the LTR-RT insertion time

Sun Jan 29 03:13:35 +01 2023 Preparing R plots files

Sun Jan 29 03:13:41 +01 2023 LTR-RT insertion time plots done

Sun Jan 29 03:13:41 +01 2023 LTR-RT-gene chimeras started

Sun Jan 29 03:13:52 +01 2023 LTR-RT-gene chimeras done

Sun Jan 29 03:13:52 +01 2023 LTR-RT near genes started

Sun Jan 29 03:14:07 +01 2023 LTR-RT near genes done

Sun Jan 29 03:14:07 +01 2023 Visualization of gene density and LTR-RTs across chromosomes

Sun Jan 29 03:15:07 +01 2023 MegaLTR Done, The results saved in (Results/Solanum_pennellii/Collected_Files)

Sorghum bicolor

#############################################

############## MegaLTR v2.0 ##############

#############################################

Contributors: Morad M Mokhtar, Achraf El Allali

Parameters: -A 3 -F /Sorghum_bicolor/Sorghum_bicolor.fna -G /Sorghum_bicolor/Sorghum_bicolor.gff -T Sorghum_bicolor_trna.fa -P Sorghum_bicolor -l 100 -L 7000 -d 1000 -D 15000 -S 85 -M 20 -B rexdb -C 20 -V 0.001 -Q 80-80-80 -E rexdb -R 0.000000013 -U 1000 -X 1000 -W 1000000 -N 9 -t 56

Check the FASTA File format.

Sun Jan 29 03:15:08 +01 2023 Start time

Sun Jan 29 03:15:30 +01 2023 LTR_FINDER & LTR_HARVEST Started

Sun Jan 29 03:50:45 +01 2023 LTR_FINDER & LTR_HARVEST Done

Sun Jan 29 03:50:46 +01 2023 LTRdigest Started

Sun Jan 29 04:03:32 +01 2023 LTRdigest Done

Sun Jan 29 04:03:32 +01 2023 TEsorter Started

Sun Jan 29 04:15:53 +01 2023 TEsorter Done

Sun Jan 29 04:15:53 +01 2023 Filtering TEsorter results Started

Sun Jan 29 04:15:53 +01 2023 Mergeing of LTR_retriever, LTRdigest, and TEsorter results

Sun Jan 29 04:17:03 +01 2023 Calculation of the LTR-RT insertion time

Sun Jan 29 04:39:20 +01 2023 Preparing R plots files

Sun Jan 29 04:39:27 +01 2023 LTR-RT insertion time plots done

Sun Jan 29 04:39:27 +01 2023 LTR-RT-gene chimeras started

Sun Jan 29 04:40:26 +01 2023 LTR-RT-gene chimeras done

Sun Jan 29 04:40:26 +01 2023 LTR-RT near genes started

Sun Jan 29 04:41:30 +01 2023 LTR-RT near genes done

Sun Jan 29 04:41:30 +01 2023 Visualization of gene density and LTR-RTs across chromosomes

Sun Jan 29 04:43:25 +01 2023 MegaLTR Done, The results saved in (Results/Sorghum_bicolor/Collected_Files)

*Trifolium pratense*

#############################################

############## MegaLTR v2.0 ##############

#############################################

Contributors: Morad M Mokhtar, Achraf El Allali

Parameters: -A 3 -F /Trifolium_pratense/Trifolium_pratense.fna -G /Trifolium_pratense/Trifolium_pratense.gff -T Trifolium_pratense_trna.fa -P Trifolium_pratense -l 100 -L 7000 -d 1000 -D 15000 -S 85 -M 20 -B rexdb -C 20 -V 0.001 -Q 80-80-80 -E rexdb -R 0.000000015 -U 1000 -X 1000 -W 1000000 -N 9 -t 56

Check the FASTA File format.

Sun Jan 29 05:36:50 +01 2023 Start time

Sun Jan 29 05:37:05 +01 2023 LTR_FINDER & LTR_HARVEST Started

Sun Jan 29 05:49:23 +01 2023 LTR_FINDER & LTR_HARVEST Done

Sun Jan 29 05:49:24 +01 2023 LTRdigest Started

Sun Jan 29 05:58:24 +01 2023 LTRdigest Done

Sun Jan 29 05:58:24 +01 2023 TEsorter Started

Sun Jan 29 06:03:44 +01 2023 TEsorter Done

Sun Jan 29 06:03:44 +01 2023 Filtering TEsorter results Started

Sun Jan 29 06:03:44 +01 2023 Mergeing of LTR_retriever, LTRdigest, and TEsorter results

Sun Jan 29 06:04:24 +01 2023 Calculation of the LTR-RT insertion time

Sun Jan 29 06:12:00 +01 2023 Preparing R plots files

Sun Jan 29 06:12:06 +01 2023 LTR-RT insertion time plots done

Sun Jan 29 06:12:06 +01 2023 LTR-RT-gene chimeras started

Sun Jan 29 06:12:38 +01 2023 LTR-RT-gene chimeras done

Sun Jan 29 06:12:38 +01 2023 LTR-RT near genes started

Sun Jan 29 06:13:26 +01 2023 LTR-RT near genes done

Sun Jan 29 06:13:26 +01 2023 Visualization of gene density and LTR-RTs across chromosomes

Sun Jan 29 06:14:47 +01 2023 MegaLTR Done, The results saved in (Results/Trifolium_pratense/Collected_Files)

*Vitis vinifera*

#############################################

############## MegaLTR v2.0 ##############

#############################################

Contributors: Morad M Mokhtar, Achraf El Allali

Parameters: -A 3 -F /Vitis_vinifera/Vitis_vinifera.fna -G /Vitis_vinifera/Vitis_vinifera.gff -T Vitis_vinifera_trna.fa -P Vitis_vinifera -l 100 -L 7000 -d 1000 -D 15000 -S 85 -M 20 -B rexdb -C 20 -V 0.001 -Q 80-80-80 -E rexdb -R 0.000000015 -U 1000 -X 1000 -W 1000000 -N 9 -t 56

Check the FASTA File format.

Sun Jan 29 02:49:22 +01 2023 Start time

Sun Jan 29 02:49:35 +01 2023 LTR_FINDER & LTR_HARVEST Started

Sun Jan 29 02:59:39 +01 2023 LTR_FINDER & LTR_HARVEST Done

Sun Jan 29 02:59:40 +01 2023 LTRdigest Started

Sun Jan 29 03:05:59 +01 2023 LTRdigest Done

Sun Jan 29 03:05:59 +01 2023 TEsorter Started

Sun Jan 29 03:07:53 +01 2023 TEsorter Done

Sun Jan 29 03:07:53 +01 2023 Filtering TEsorter results Started

Sun Jan 29 03:07:53 +01 2023 Mergeing of LTR_retriever, LTRdigest, and TEsorter results

Sun Jan 29 03:08:34 +01 2023 Calculation of the LTR-RT insertion time

Sun Jan 29 03:10:24 +01 2023 Preparing R plots files

Sun Jan 29 03:10:31 +01 2023 LTR-RT insertion time plots done

Sun Jan 29 03:10:31 +01 2023 LTR-RT-gene chimeras started

Sun Jan 29 03:10:38 +01 2023 LTR-RT-gene chimeras done

Sun Jan 29 03:10:38 +01 2023 LTR-RT near genes started

Sun Jan 29 03:10:49 +01 2023 LTR-RT near genes done

Sun Jan 29 03:10:49 +01 2023 Visualization of gene density and LTR-RTs across chromosomes

Sun Jan 29 03:11:49 +01 2023 MegaLTR Done, The results saved in (Results/Vitis_vinifera/Collected_Files)

*Zea mays*

#############################################

############## MegaLTR v2.0 ##############

#############################################

Contributors: Morad M Mokhtar, Achraf El Allali

Parameters: -A 3 -F /Zea_mays/Zea_mays.fna -G /Zea_mays/Zea_mays.gff -T Zea_mays_trna.fa -P Zea_mays -l 100 -L 7000 -d 1000 -D 15000 -S 85 -M 20 -B rexdb -C 20 -V 0.001 -Q 80-80-80 -E rexdb -R 0.000000013 -U 1000 -X 1000 -W 1000000 -N 9 -t 56

Check the FASTA File format.

Sun Jan 29 01:03:05 +01 2023 Start time

Sun Jan 29 01:04:13 +01 2023 LTR_FINDER & LTR_HARVEST Started

Sun Jan 29 04:37:12 +01 2023 LTR_FINDER & LTR_HARVEST Done

Sun Jan 29 04:37:14 +01 2023 LTRdigest Started

Sun Jan 29 07:11:01 +01 2023 LTRdigest Done

Sun Jan 29 07:11:01 +01 2023 TEsorter Started

Sun Jan 29 08:23:20 +01 2023 TEsorter Done

Sun Jan 29 08:23:21 +01 2023 Filtering TEsorter results Started

Sun Jan 29 08:23:22 +01 2023 Mergeing of LTR_retriever, LTRdigest, and TEsorter results

Sun Jan 29 08:30:06 +01 2023 Calculation of the LTR-RT insertion time

Sun Jan 29 10:56:54 +01 2023 Preparing R plots files

Sun Jan 29 10:57:04 +01 2023 LTR-RT insertion time plots done

Sun Jan 29 10:57:04 +01 2023 LTR-RT-gene chimeras started

Sun Jan 29 11:02:01 +01 2023 LTR-RT-gene chimeras done

Sun Jan 29 11:02:01 +01 2023 LTR-RT near genes started

Sun Jan 29 11:11:02 +01 2023 LTR-RT near genes done

Sun Jan 29 11:11:02 +01 2023 Visualization of gene density and LTR-RTs across chromosomes

Sun Jan 29 11:19:19 +01 2023 MegaLTR Done, The results saved in (Results/Zea_mays/Collected_Files)
