# Supplementary figures and images for: MegaLTR: a web server and standalone pipeline for detecting and annotating LTR-retrotransposons in plant genomes
Source: Front Plant Sci. 2023 Sep 20;14:1237426. doi: 10.3389/fpls.2023.1237426 (PMC10552921; doi:10.3389/fpls.2023.1237426)

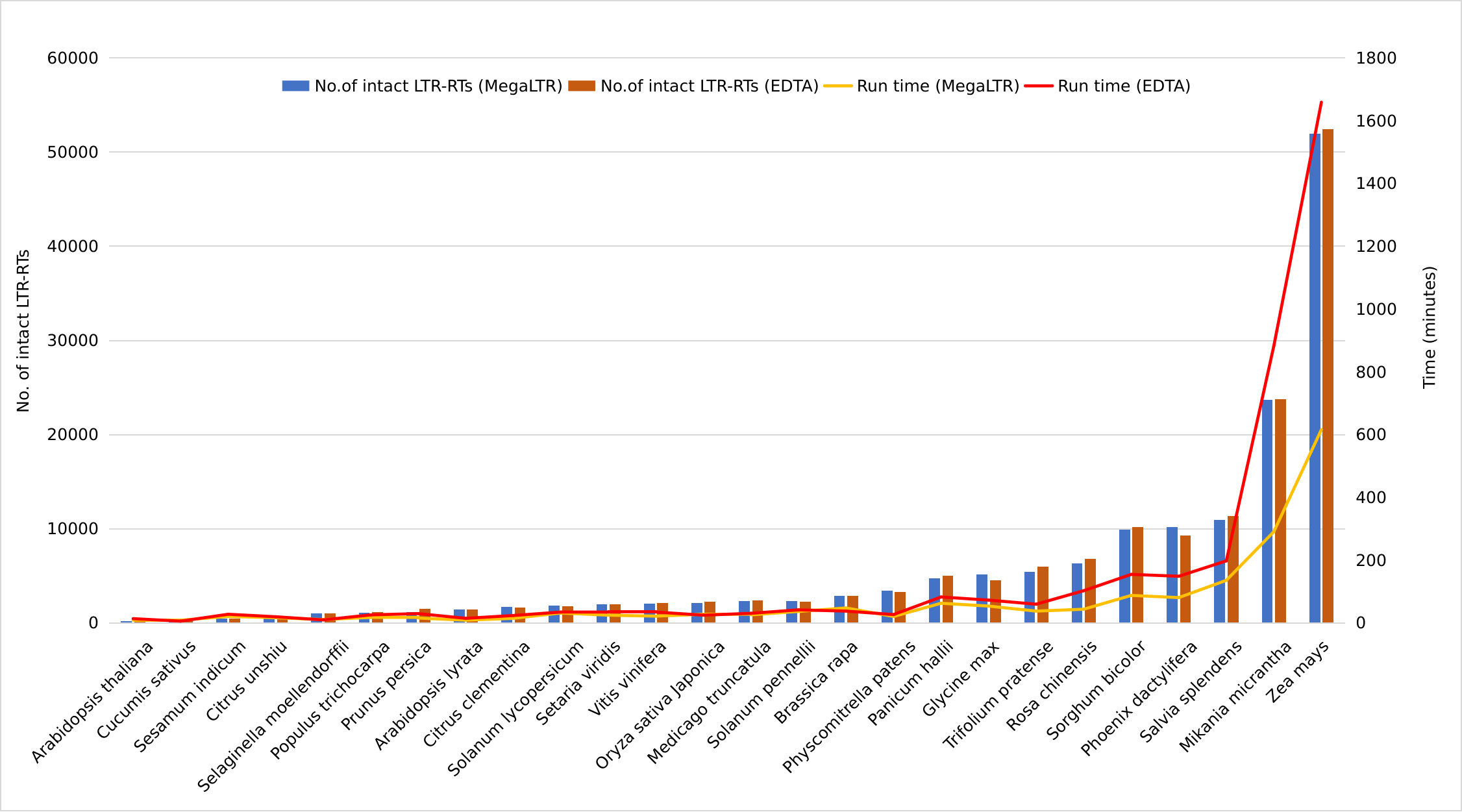

Supplement: Supplementary file 6 [file Image_1.png]

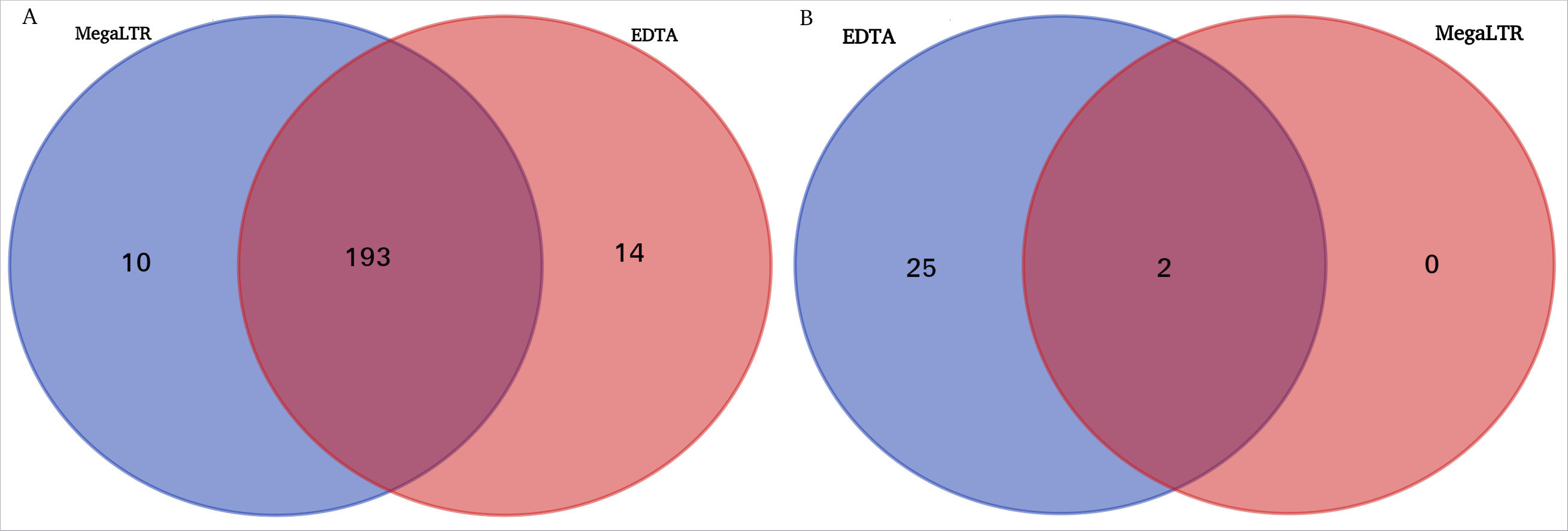

Supplement: Supplementary file 7 [file Image_2.jpeg]
